# Acute irradiation induces a senescence-like chromatin structure in mammalian oocytes

**DOI:** 10.1038/s42003-023-05641-0

**Published:** 2023-12-12

**Authors:** Claudia Baumann, Xiangyu Zhang, Muthugapatti K. Kandasamy, Xiaohan Mei, Shiyou Chen, Kayvan F. Tehrani, Luke J. Mortensen, Wendy Watford, Ashley Lall, Rabindranath De La Fuente

**Affiliations:** 1 Department of Physiology and Pharmacology, College of Veterinary Medicine, University of Georgia, Athens, GA USA; 2 Regenerative Biosciences Center (RBC), University of Georgia, Athens, GA USA; 3 Department of Genetics University of Georgia, Athens, GA USA; 4 Division of Surgical Research, University of Missouri, School of Medicine, Columbia, MO USA; 5 School of Chemical, Materials and Biomedical Engineering, University of Georgia, Athens, GA USA; 6 Department of Infectious Diseases, University of Georgia, Athens, GA USA; 7 Present Address: Weill Cornell Medical College, New York, NY USA; 8 Present Address: University of Illinois at Urbana-Champaign, Urbana, IL USA

**Keywords:** Senescence, Centromeres

## Abstract

The mechanisms leading to changes in mesoscale chromatin organization during cellular aging are unknown. Here, we used transcriptional activator-like effectors, RNA-seq and superresolution analysis to determine the effects of genotoxic stress on oocyte chromatin structure. Major satellites are organized into tightly packed globular structures that coalesce into chromocenters and dynamically associate with the nucleolus. Acute irradiation significantly enhanced chromocenter mobility in transcriptionally inactive oocytes. In transcriptionally active oocytes, irradiation induced a striking unfolding of satellite chromatin fibers and enhanced the expression of transcripts required for protection from oxidative stress (*Fermt1, Smg1*), recovery from DNA damage (*Tlk2, Rad54l*) and regulation of heterochromatin assembly (*Zfp296, Ski-oncogene*). Non-irradiated, senescent oocytes exhibit not only high chromocenter mobility and satellite distension but also a high frequency of extra chromosomal satellite DNA. Notably, analysis of biological aging using an oocyte-specific RNA clock revealed cellular communication, posttranslational protein modifications, chromatin and histone dynamics as the top cellular processes that are dysregulated in both senescent and irradiated oocytes. Our results indicate that unfolding of heterochromatin fibers following acute genotoxic stress or cellular aging induced the formation of distended satellites and that abnormal chromatin structure together with increased chromocenter mobility leads to chromosome instability in senescent oocytes.

## Abstract (web summary)

Super-resolution imaging and RNA clock analyses show that exposure of mouse oocytes to ionizing radiation compromises chromatin organization and dramatically accelerates biological aging.

## Introduction

In mammalian cells, pericentric heterochromatin is essential for higher-order chromosome organization, nuclear architecture, and maintenance of genome integrity^1,2^. Heterochromatin domains exhibit profound conformational changes during cellular senescence with dire consequences for genome integrity, but little is known about the underlying mechanisms^2,3^. Senescent cells undergo dramatic changes in genome organization, potentially altering chromatin conformation and chromosome packaging at different scales^4,5^. For example, chromatin conformation (Hi-C) assays revealed that both senescent human fibroblasts and Hutchinson-Gilford progeria cells exhibit a relaxation of chromatin topographical organization with a reduction of local interactions between topologically associated domains (TADs) in the human genome^5^^,^^6,7^.

Senescence-associated distension of satellite DNA (SADs) is an early senescence marker consistently detected in human and mouse models of cellular aging, suggesting that heterochromatin reorganization may be an integral component of the senescence process^3,4^. Elegant studies suggest that SADs formation represents a large-scale unfolding of chromatin, above the level of the nucleosome^4^. However, the mechanisms involved in regulating such striking changes in mesoscale chromatin structure are not clear at present. In contrast with our current understanding of nucleosome organization in the human and mouse genomes^6–8^, little is known about the mechanisms that regulate mesoscale chromatin organization from the canonical 10 nm fiber into a highly compact metaphase chromosome. Importantly, the regulation of some of the most critical levels of large-scale chromatin organization, as well as the response of centromeric heterochromatin domains to developmental or environmental transitions remains poorly understood.

Direct visualization of 3D-genome organization in space and time and with a level of resolution beyond the optical diffraction limit is essential to understand the biological principles of chromatin organization, chromatin fiber folding, and chromosome compaction in the mammalian genome. Functional differentiation of chromatin structure in pre-ovulatory oocytes provides a prominent example of large-scale chromatin remodeling with direct implications for chromosome stability and developmental potential^9,10^. In fully-grown oocytes at the germinal vesicle (GV) stage, chromatin is initially found in a highly decondensed state to sustain high levels of nascent transcription, a configuration called non-surrounded nucleolus (NSN)^11,12^. However, in preparation for meiotic resumption, oocytes undergo a striking change in nuclear organization with formation of a perinucleolar chromatin rim or karyosphere, a configuration known as surrounded nucleolus (SN)^10,11^. Chromatin remodeling into the SN configuration is also associated with a concomitant global repression of nascent transcription^9,12^. Importantly, these critical developmental transitions in chromatin organization are highly conserved between human and mouse ova^13^. The large size of the mammalian oocyte nucleus and the potential to analyze chromatin in the presence or absence of transcription provides a natural in vivo model to dissect the molecular pathways of chromatin remodeling at critical developmental milestones.

Here, we use transcriptional activator-like effectors (TALEs) against major satellite sequences^14^ for live-cell chromocenter tracking, superresolution structured illumination^15^, and RNA sequencing to determine the effects of genotoxic stress induced by acute γ-irradiation or reproductive senescence on large-scale chromatin organization. Our results indicate that in transcriptionally inactive (SN) oocytes, chromocenters are extremely mobile and dynamically interact with the nucleolus. Both acute irradiation and cellular aging significantly increased chromocenter mobility in SN oocytes but resulted in a striking distention of satellite DNA in transcriptionally active (NSN) oocytes. Notably, senescent oocytes exhibit not only severe distension of satellite DNA but also a high frequency of extra chromosomal DNA fragments. Our results indicate that both increased chromocenter mobility and abnormal chromatin structure contribute to the mechanisms of chromosome instability in senescent oocytes.

## Results

### Live-cell imaging of major satellite DNA and chromocenter formation in the oocyte genome

**Fig. 1:**
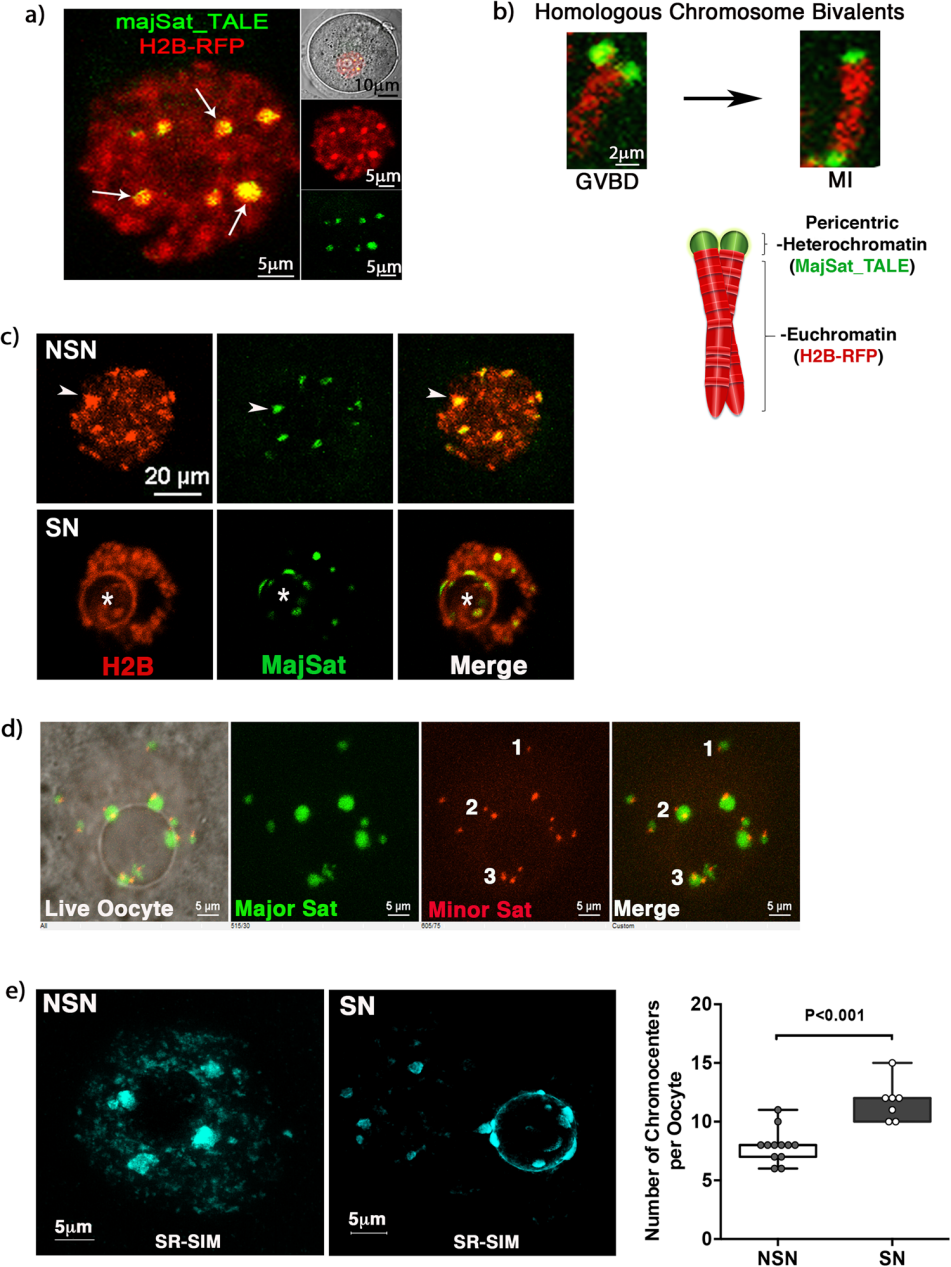
Live-cell imaging of major satellite DNA using fluorescent TALEs. **a** Germinal vesicle stage oocyte microinjected with a transcriptional activator-like effector (majSat_TALE). Major satellite sequences (arrows, green) cluster into large chromocenters. Histone H2B-RFP (red). **b** MajSat_TALE reveals the physical proximity of pericentric heterochromatin domains from a homologous chromosome bivalent after germinal vesicle breakdown (GVBD) and their polar orientation at the metaphase-I (MI) stage. **c** Distinct chromocenter distribution in NSN and SN oocytes. Live cell imaging and confocal microscopy indicate that NSN oocytes exhibit chromocenters (green) distributed throughout the nucleoplasm (red) including the nuclear periphery (arrowheads). In SN oocytes, most chromocenters become associated with the nucleolus (asterisk). **d** Microinjection of majSat_TALE to detect pericentric heterochromatin domains (green) and MinSat_TALE to detect minor satellite sequences at the centromere (red) revealed that small chromocenters are formed by a single homologous chromosome bivalent with fused centromeres whereas large chromocenters are formed by the interaction and coalescence of pericentric heterochromatin domains from 2 or 3 homologous chromosome bivalents. **e** Superresolution structured illumination (SR-SIM) confirmed the presence of large chromocenters in NSN oocytes and revealed a significant (*p* = 0.0002) increase in the number of chromocenters in SN oocytes compared to NSN oocytes in (*n* = 7) and (*n* = 12) biologically independent samples, respectively. Box and whiskers plot with minimum, maximum and 25th and 75th percentiles. Mann–Whitney Test with a Cohen’s *d* effect size of 2.23 and actual confidence intervals of the median of 96.14% (NSN) and 98.44% (SN). Shown are maximum intensity projections of the entire oocyte nucleus stained with DAPI and pseudo colored in cyan for contrast.

Pericentric heterochromatin formation is essential for maintenance of chromosome stability during female meiosis^16,17^. In the mouse genome, pericentric heterochromatin (PCH) domains are formed by 6 megabases of repetitive major satellite DNA adjacent to the centromere of each chromosome^18^. However, little is known about the role of PCH in the regulation of large-scale chromatin organization and nuclear architecture. Microinjection of a TALE-effector^14^ into live, GV stage oocytes revealed that major satellite sequences (green) aggregate into large globular domains or chromocenters in oocytes that exhibit the NSN configuration (Fig. 1a). Simultaneous examination of histone H2B (red) revealed that in addition to its widespread nucleoplasmic localization, histone H2B exhibits enhanced fluorescence at chromocenters where it is co-localized with the major satellites (Fig. 1a; arrows), consistent with the formation of a highly compact heterochromatin domain. Live-cell imaging during germinal vesicle break down (GVBD) indicates that the presence of large chromocenters is due to the coalescence of PCH domains from homologous chromosome bivalents (Fig. 1b and Supplementary Figure 1a). Paired homologous chromosome bivalents can be detected 2.5 h after GVBD where the major satellite sequences (green) of each bivalent are found in close apposition (Supplementary Figure 1a). In contrast, the PCH domains from each bivalent exhibit a polar orientation at the MI stage (Fig. 1b). In oocytes that exhibit the NSN configuration, large chromocenters are distributed throughout the nucleoplasm including the nuclear periphery (Fig. 1c; arrowhead). However, live cell imaging revealed continuous oscillatory movements in which some adjacent chromocenters can establish physical interactions (Supplementary Movie 1). In SN oocytes, most chromocenters become associated with the nucleolus (Fig. 1c). Notably, these interactions are highly dynamic as chromocenters may fuse with each other and subsequently split before establishing a nucleolar association followed by a transient detachment from the nucleolar surface (Supplementary Movie 2). These results indicate that SN oocytes exhibit high chromocenter mobility and suggest that chromocenter formation may be the result of dynamic interactions between major satellites from several homologous chromosome bivalents.

To determine the number of homologous chromosome bivalents that form each chromocenter in NSN oocytes, we microinjected TALE-effectors for both major (green) and minor (red) satellite sequences, which label PCH domains and centromeres, respectively (Fig. 1d). Detection of minor satellites with confocal microscopy revealed the localization of centromeres that appeared fused as indicated by a single red signal adjacent to major satellite DNA (green) on an individual homologous chromosome bivalent (Fig. 1 d1; Merge). Super resolution structural illumination (SR-SIM)^15^ and 3D spatial analysis confirmed the presence of fused centromeres on each homologous chromosome bivalent and revealed chromocenters with one or two minor satellite signals (Supplementary Figure 1b). This indicates that the large chromocenters in NSN oocytes are formed by the association of major satellites from more than one homologous chromosome bivalent, as determined by the number of fused minor satellite signals (Fig. 1 d2; Merge; Supplementary Figure 1b, d and Supplementary Movie 3), while smaller chromocenters are formed by a single homologous chromosome bivalent with fused centromeres as reflected by a single minor satellite signal (Supplementary Figure 1b, c and Supplementary Movie 4). These results indicate that major satellites from different homologous chromosome bivalents coalesce to form the large chromocenters present in NSN oocytes. Consistent with these results, SR-SIM revealed an average of 7.9 ± 1.4 chromocenters in oocytes with the NSN configuration confirming that major satellites from 2–3 homologous chromosome pairs may coalesce to form a large chromocenter (Fig. 1e and Supplementary Figure 1). Importantly, in SN oocytes the average number of chromocenters increased to 11.7 ± 1.7 (*p* < 0.001) indicating that at this stage, most chromocenters are formed by interactions between two homologous chromosome bivalents but also occasionally by a single homologous chromosome bivalent (Fig. 1e). These results indicate that chromocenter organization is highly dynamic during the transition into the SN configuration in preparation for meiotic resumption.

**Fig. 2:**
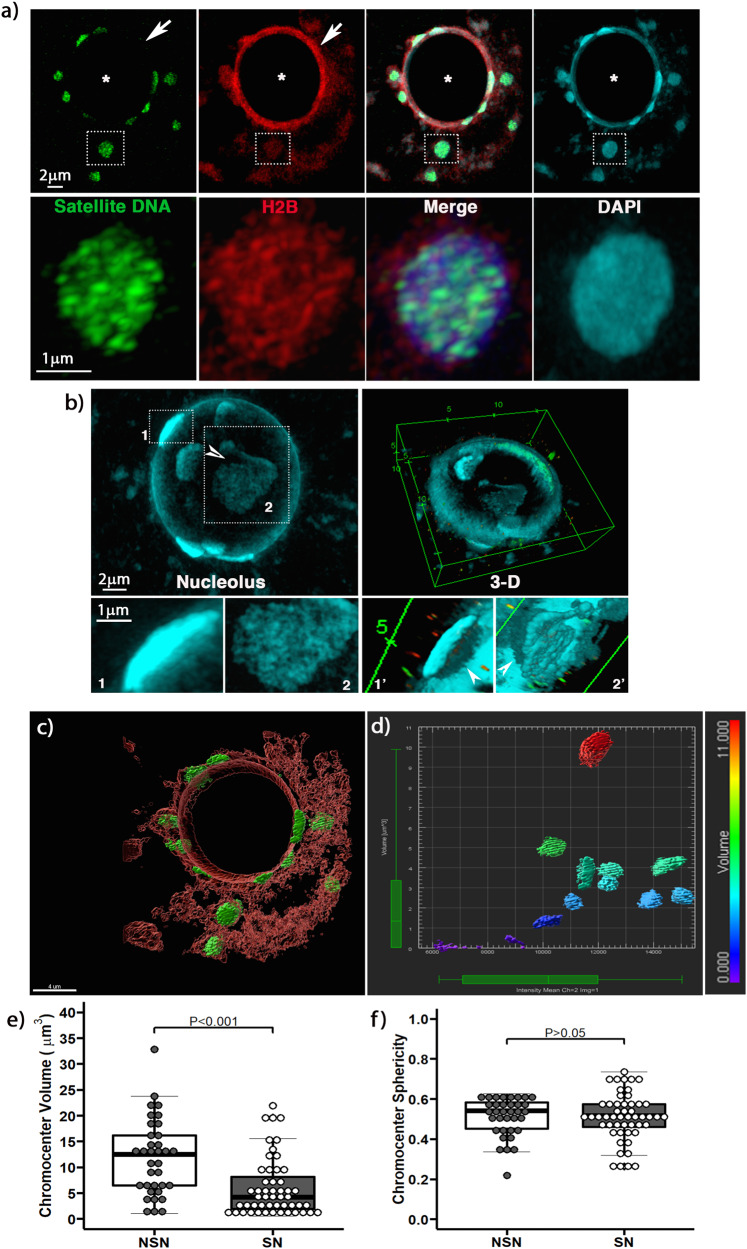
Superresolution analysis of major satellite DNA and chromocenter organization in the oocyte genome. **a** Superresolution structured illumination resolves a network of densely packed major satellite chromatin fibers (green) within compact chromocenters (inset). Histone H2B (red). DAPI is shown in Cyan. Scale bar = 2 μm. **b** Reconstruction of the oocyte nucleolus using 3D-SIM. Chromocenters show differences in size and topographical organization (insets 1–2). 3D-renderings reveal chromocenter splitting (arrowhead) and chromatin fibers interacting with the nucleolar surface (insets 1’–2’; arrowheads). Scale bar = 2 μm. **c** Quantification of chromocenter volume using a 3-D rendering of superresolved Z stack images of the entire nucleus of an SN oocyte. Major satellite DNA (green), histone H2B (red). (*) Demarcates the nucleolus. Scale bar = 4 μm. **d** Quantitative volumetric analysis was conducted using the 3-D surface renderings of each chromocenter. Differences in volume (μm^3^) are indicated according to the color-coded volumetric scale bar. **e** Analysis of the average chromocenter volume in NSN oocytes (*n* = 12) and SN oocytes (*n* = 7) of biologically independent samples (*P* = 0.000203). **f** Similar chromocenter sphericity indicates that chromocenters form globular structures in both NSN and SN oocytes (*P* = 0.9297). Box plots indicate the median, minimum and maximum values as well as the 25th and 75th percentiles. T-Test with a Cohen’s *d* effect size of 0.91 (E) and −0.22 (F) and actual 95% confidence intervals of 2.88 and 8.78 (E) and −0.05 and 0.04 (F), respectively.

Next, to gain insight into the basic principles of large-scale satellite chromatin fiber folding and its organization into chromocenters, we conducted a serial analysis of superresolved images on the Z plane^15^. SR-SIM resolves the mesoscale organization of major satellite DNA beyond the optical diffraction limit and reveals a network of tightly packed chromatin fibers (green) that also exhibit bright H2B signal (red) (Fig. 2a; Inset). In oocytes with the SN configuration, most chromocenters are attached to the nucleolus and are highly condensed (Fig. 2a). Notably, the perinucleolar rim is formed by chromatin fibers labeled exclusively with H2B (Fig. 2a; arrow). This suggests that chromocenters remain as separate PCH domains, even after their anchoring to the nucleolus, and that karyosphere formation is completed by the surrounding of the nucleolus with H2B-labeled chromatin.

3D-reconstruction with SR-SIM revealed differences in chromocenter topographical organization at the nucleolar surface (Fig. 2b; Inset 1 and 2) and resolved a tightly packed network of major satellite DNA fibers within chromocenters (Fig. 2b; Inset 2). In addition, some chromocenters might split while still attached to the nucleolus (Fig. 2b; Inset 2; arrowhead). These results provide additional evidence that chromocenters remain as individual, distinct PCH domains that actively interact with the nucleolus. Three-dimensional renderings also revealed striking protrusions of a network of chromatin fibers (Inset 1’ and 2’; arrowheads) interacting with the nucleolar surface (Fig. 2b and Supplementary Movie 5). Quantitative, computational image analysis of individual chromocenters using 3D-renderings and surface segmentation with IMARIS (Fig. 2c) indicates that in SN oocytes, chromocenters range in size from 2–10 cubic microns (Fig. 2d). Importantly, comparison of the average chromocenter volume between NSN oocytes (11.8 ± 7.2 μm^3^) and SN (6.04 ± 5.6 μm^3^) oocytes revealed a significant reduction (*P* < 0.001) in chromocenter volume in SN oocytes (Fig. 2e). No significant differences were detected in chromocenter sphericity (Fig. 2f), suggesting that chromocenters form globular structures in NSN as well as SN oocytes. Both, the increased chromocenter number and a significantly reduced volume are consistent with the formation of chromocenters from one or two homologous chromosome bivalents in SN oocytes. Together with the chromocenter fusions and splitting events observed in live oocytes, these results suggest extensive rearrangements of interactions between major satellites of different homologous chromosome bivalents in preparation for meiotic resumption.

### Acute irradiation and oocyte aging induce enhanced chromocenter mobility

**Fig. 3:**
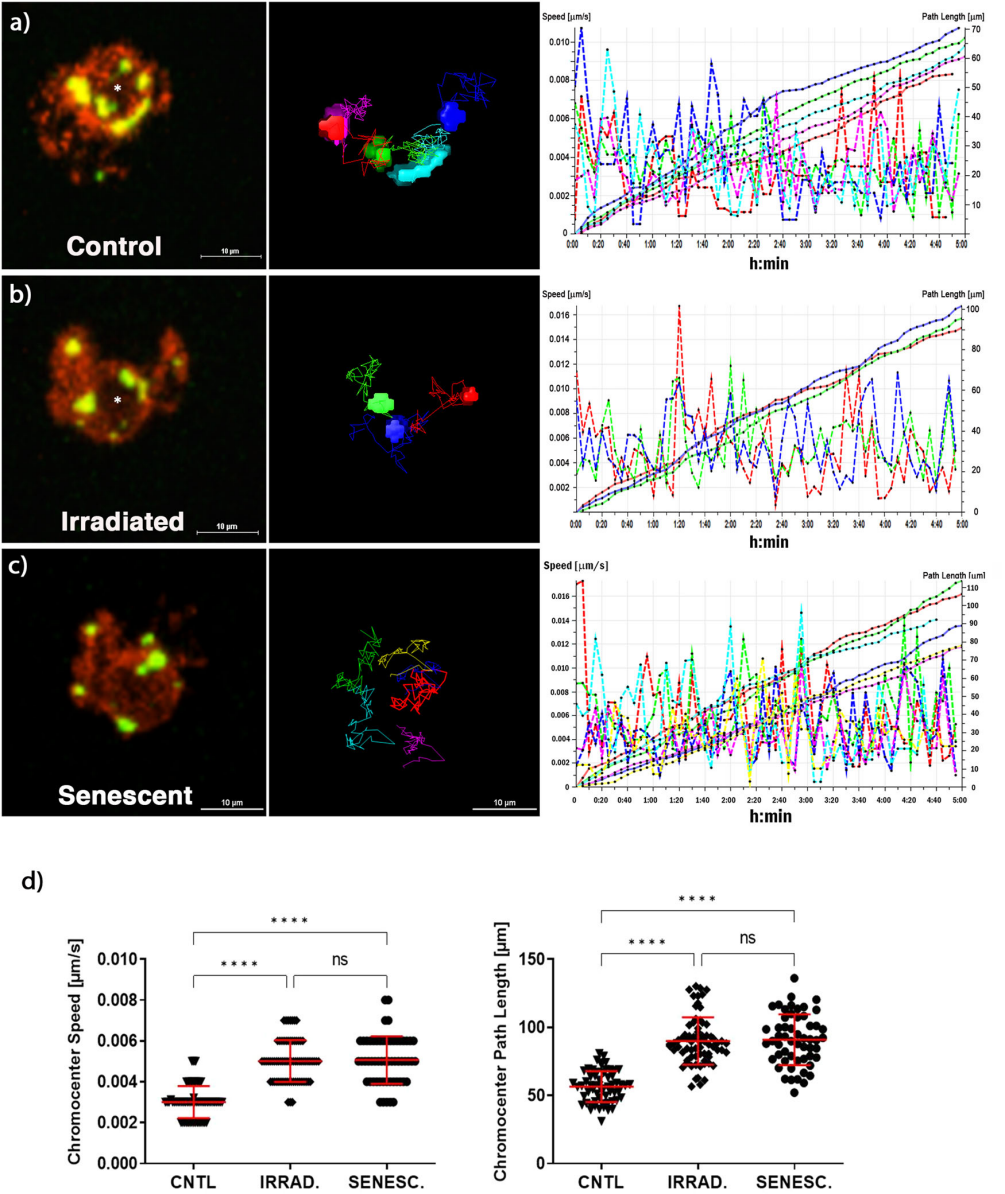
γ-Irradiation increases chromocenter mobility in the oocyte genome. **a** Live-cell analysis of chromocenter dynamics in control SN oocytes using majSat-TALE (green), Histone H2B (red). (*) Demarcates the position of the nucleolus. Speed (μm/s) and path length (μm) were quantified over a period of 5 h. **b** γ-Irradiation increased chromocenter mobility and induced detachment from the nucleolus. **c** Non-irradiated senescent oocytes exhibit inherently high chromocenter mobility. **d** Average mobility patterns observed in chromocenters from young control oocytes (*n* = 11), young, irradiated oocytes (*n* = 10) and non-irradiated, senescent oocytes (*n* = 11). Data presented as the mean ± S.D. of three independent experimental replicates. One-way ANOVA with Tukey’s multiple comparisons test with ****P < 0.0001 and *P* = 0.9997 for ns (not significant) for chromocenter speed and ****P < 0.0001 and *P* = 0.9421 for ns (not significant) for chromocenter path length. 95% confidence intervals were −0.002415 to −0.001602 (CNTL versus IRRAD.), −0.002445 to −0.001580 (CNTL vs. SENESC.), and −0.0004204 to 0.0004122 (IRRAD. versus SENESC.) for chromocenter speed. For chromocenter path length, 95% confidence intervals were −40.18 to −26.79 (CNTL versus IRRAD.), −41.57 to −27.31 (CNTL vs. SENESC.), and −7.816 to 5.905 (IRRAD. versus SENESC.) for chromocenter speed.

To determine the effect of acute or persistent genotoxic stress on chromocenter mobility, we exposed young (1-month old) SN oocytes to a 5 Gy dose of acute γ-irradiation and compared chromatin dynamics with non-irradiated, senescent (>10-month-old) oocytes. Live-cell imaging revealed that chromocenters in control young, non-irradiated SN oocytes exhibit a path length of 55–71 microns in a 5 h period (Fig. 3a and Supplementary Movie 6). In contrast, acute irradiation induced a significant increase in chromocenter mobility that changed the pattern of oscillatory movements at the nucleolus into a linear displacement following detachment of chromocenters from the nucleolar surface, resulting in a total path length of 90–101 microns (Fig. 3b and Supplementary Movie 7). The average speed (0.003 μm/s) after tracking 60 chromocenters in control oocytes (*n* = 11) increased significantly (*p* < 0.0001) to 0.005 μm/s in 71 chromocenters tracked in irradiated oocytes (*n* = 10), resulting in a mean total path length of 56.5 microns in controls and 89.9 microns in irradiated oocytes (Fig. 3d). Notably, analysis of (*n* = 55) chromocenters in non-irradiated senescent oocytes (*n* = 11) revealed high chromocenter mobility resulting in a total path length of 76–115 microns. The average chromocenter speed (0.005 μm/s) in aged oocytes showed no significant differences with that observed in irradiated, young oocytes (Fig. 3c, d), resulting in an average total path length of 90.9 microns in senescent oocytes. These results indicate that non-irradiated senescent oocytes exhibit inherently high chromocenter mobility.

The chromatin remodeling protein ATRX associates with highly condensed, DAPI-bright chromocenters in GV stage oocytes (Supplementary Figures 2a–d) and is required for centromere stability in mouse oocytes^16,19^. SR-SIM analysis revealed that γ-irradiation results in a striking chromocenter decondensation after 24 h in oocytes that exhibit the NSN configuration, Notably, ATRX remained bound to decondensed chromocenter chromatin fibers in irradiated oocytes (Supplementary Figure 2e and Supplementary Figures 3a–c).

### γ-Irradiation induces distension of satellite DNA and abnormal chromatin structure in transcriptionally active (NSN) oocytes

**Fig. 4:**
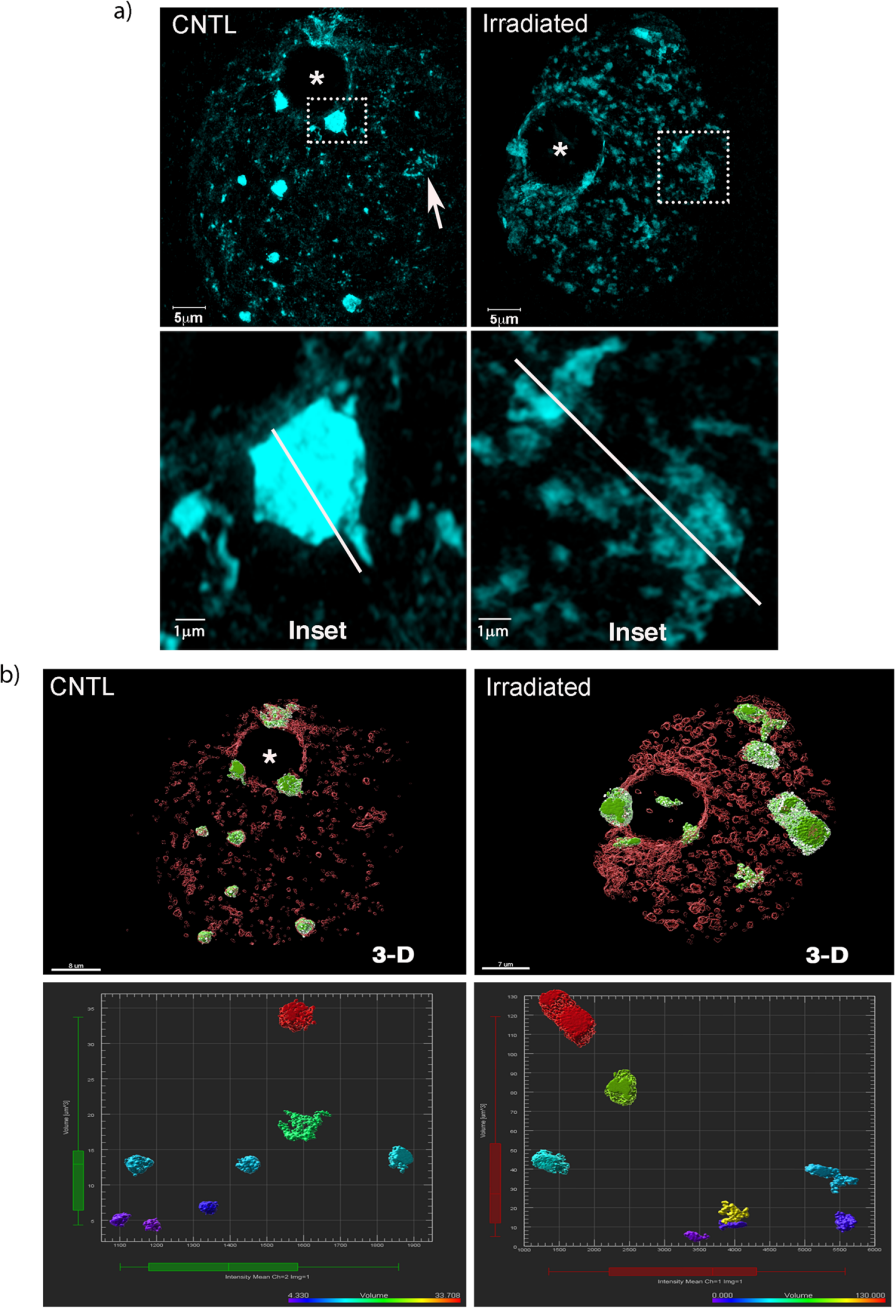
Distention of major satellite DNA sequences and abnormal chromatin fiber folding in response to γ-irradiation. **a** Structured illumination of control and irradiated oocytes 24 h following γ-irradiation. DNA is stained with DAPI (Cyan). Non-irradiated oocytes exhibit highly condensed chromocenters with DAPI-bright staining (Inset). Scale bar = 5 μm. In contrast, irradiated oocytes exhibit a striking decondensation of chromocenters (Inset). Scale bar= 5 μm. Note the separation of chromatin fibers in a cross-section of the chromocenter (Inset; Line scan). **b** 3D-reconstruction of SR-SIM images to quantify chromocenter volume (μm^3^) and fluorescence intensity in control and irradiated oocytes. Major satellite DNA sequences (green); histone H2B (red). (*) Demarcates the nucleolus.

Next, we quantified the extent of chromocenter decondensation and potential changes in chromatin fiber folding induced by γ-irradiation. We used SR-SIM to analyze single 91 nanometer scans (on the Z axis) of the nucleus of NSN oocytes prior to and 24 h after irradiation. In control non-irradiated oocytes, euchromatin is organized into a lose network of DNA fibers that is widely distributed and occupies the entire volume of the germinal vesicle (Fig. 4a). Maximum intensity projections of SR-SIM scans resolve tracks and twists of fibers (Fig. 4a; arrow) resembling the large-scale mid prophase chromatid folds previously described in mouse fibroblasts^20^. In contrast, heterochromatin is organized into a tightly packed network of major satellite DNA fibers forming several DAPI-bright, compact chromocenters (Fig. 4a; Inset and Supplementary Figure 2). Notably, chromocenters exhibit a striking distension 24 h following irradiation, characterized by unfolding of heterochromatin fibers and a subsequent increase in fiber–fiber distance (Fig. 4a: Insets, Line Scan and Supplementary Figure 3).

Quantitative analysis of chromocenter volume using 3D-SIM and surface segmentation with IMARIS based on DAPI intensity and TALE major satellite labeling (green) revealed that the volume of chromocenters in control NSN oocytes ranges between 3 to 35 μm^3^ (Fig. 4b and Supplementary Movie 8). However, γ-irradiation induced a significant increase in chromocenter volume with some chromocenters reaching between 50–130 μm^3^ (Fig. 4b and Supplementary Movie 9). Moreover, we observed a significant increase in the average chromocenter volume and a reduction in chromocenter sphericity 24 h following irradiation, consistent with distension of heterochromatin fibers and loss of chromocenter compaction (Supplementary Figure 4a). These results indicate that the striking changes in mesoscale chromatin organization induced by γ-irradiation are due to abnormal packing of large-scale heterochromatin fibers, disruption of major satellite chromatin fiber folding and a subsequent increase in fiber–fiber distance within disrupted chromocenters. Importantly, our results also indicate that genotoxic stress is a contributing factor inducing distension of major satellites in NSN oocytes.

**Fig. 5:**
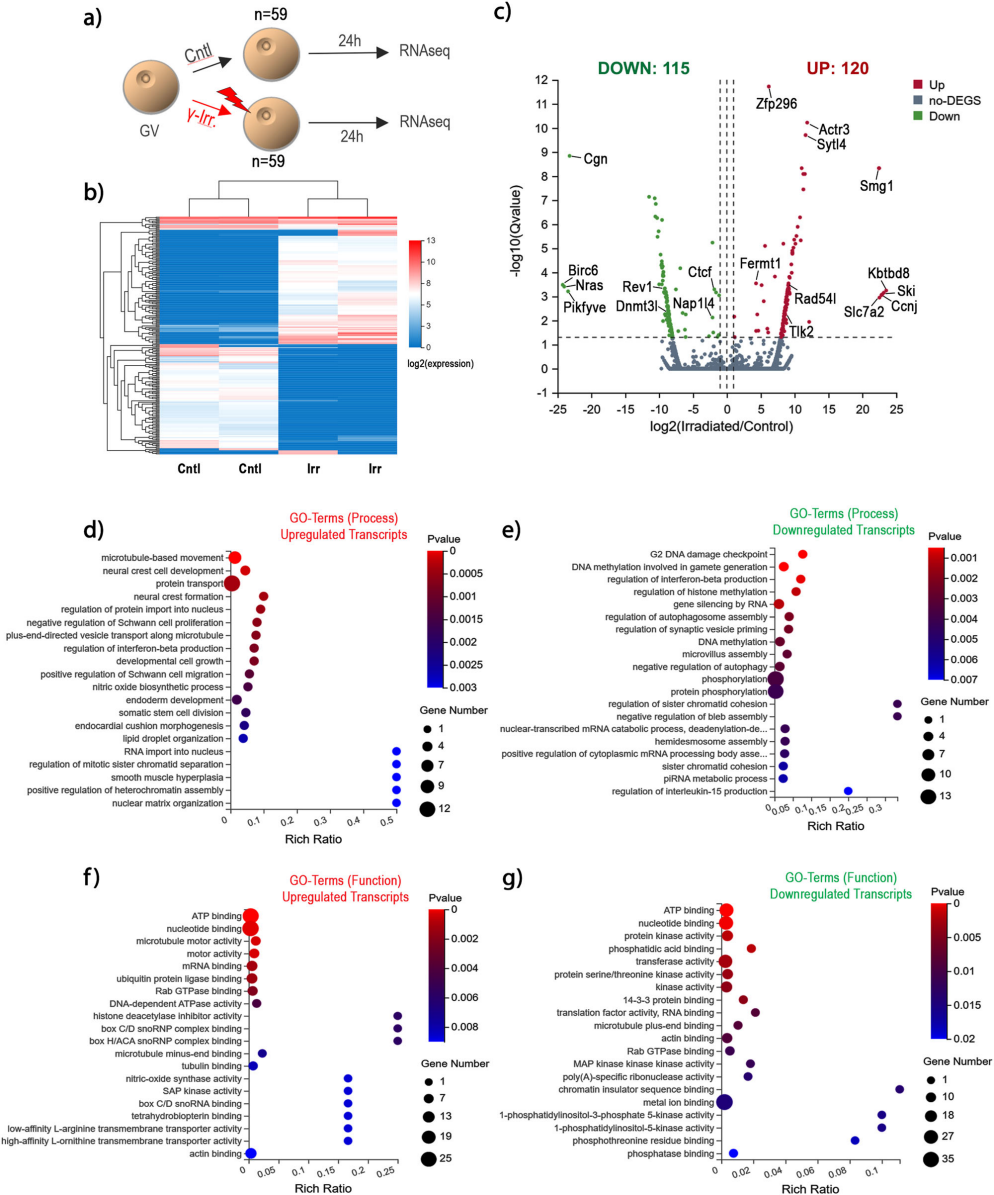
Transcriptome analysis following γ-irradiation of NSN oocytes. **a** Germinal vesicle (GV) stage oocytes on day 16 of post-natal development were exposed to γ-irradiation (5 Gy) in *n* = 2 independent biological experiments. **b** Heatmap generated from transcriptional changes detected in control (*n* = 59) versus γ-irradiated (*n* = 59) oocytes, respectively 24 h post-irradiation in two replicates. Color shading indicates changes in log2 expression values. **c** Volcano plot of Control-vs-Irradiated transcript levels (DEseq2 method) depicting the log2-fold change of differential transcript expression. The Y axis represents log10 significance value. Upregulated transcripts (red); downregulated transcripts (green). **d** Overrepresented GO-Terms (biological process) for upregulated transcripts. Shading indicates level of significance. Size of the bubble represents number of transcripts in category. **e** Overrepresented GO-Terms (biological process) for downregulated transcripts. **f** Overrepresented GO-Terms (molecular function) for upregulated transcripts. **g** Overrepresented GO-Terms (molecular function) for downregulated transcripts.

To gain molecular insight into the mechanisms of chromocenter decondensation we conducted a transcriptome analysis of young oocytes 24 h following γ-irradiation (Fig. 5a–c and Supplementary Figure 5). Denuded oocytes were collected on day 16 of post-natal development, a stage at which >90% exhibit the NSN configuration and are transcriptionally active^10^. Analysis of differentially expressed transcripts revealed significantly (*P* < 0.05) enhanced expression of 120 transcripts and a significant downregulation of 115 transcripts 24 h after irradiation compared with control non-irradiated oocytes. Notably, the zinc finger protein (*Zfp296*) exhibited the highest statistical significance and showed a 6.1-fold overexpression in irradiated oocytes. Zfp296 is a component of mouse PCH that interacts with heterochromatin proteins, including ATRX and contributes to the regulation of histone modifications at major satellite repeats^21^. In addition, the actin-related protein 3 (*Actr3*) which functions in F-actin polymerization, nuclear lamina assembly and chromatin reorganization^22^ exhibited a significant (*p* < 0.05) 11.8-fold overexpression. *Kbtbd8*, a ubiquitin ligase involved in free-radical control and oocyte quality^23^, the *Ski proto-oncogene*, which directly binds to and is required to regulate histone modifications at major satellites^24^ and the solute carrier (*Slc7a2*), that potentially interacts with the Ski proto-oncogene^24^ were all upregulated >20 fold in irradiated oocytes (*p* < 0.05). Moreover, transcripts required for protection from oxidative stress (*Fermt1, Smg1*)^25,26^ and recovery from DNA damage (*Tlk2, Rad54l*)^27,28^ showed a significant (*P* < 0.05) overexpression. In contrast, amongst the most significantly downregulated transcripts, cingulin (*Cgn*) a tight junction protein known to cause activation of cellular senescence pathways following its functional ablation^29^, *Birc6/Bruce*, an inhibitor of apoptosis^30^ that regulates DNA double-strand break response^31^ and is required for genome stability^32^ as well as the N-Ras proto-oncogene, which is required to modulate the DNA damage response^33^ were all downregulated >20 fold. In addition, *Rev1* a translesion synthesis protein that regulates radiation resistance^34^ and Dnmt3l were downregulated >5 fold. Gene ontology (GO) analysis of upregulated transcripts indicates roles in cellular processes such as RNA import into nucleus, regulation of mitotic sister chromatid separation, positive regulation of heterochromatin assembly and nuclear matrix organization while GO terms for cellular function revealed a role in histone deacetylase inhibitor activity, box C/D snoRNP complex binding, box H/ACA snoRNP complex binding and nitric oxide synthase activity (Fig. 5d, f). In contrast, GO analysis for downregulated transcripts revealed roles in cellular processes such as G2 DNA damage checkpoint and regulation of histone methylation while cellular functions involved chromatin insulator sequence binding, phosphatidyl inositol kinase activity and phospho threonine residue binding (Fig. 5e, g). Importantly, the overexpression or downregulation of several randomly selected transcripts from the RNA-seq dataset were validated in an independent experiment using real-time PCR (Supplementary Figure 4b). These results indicate that the effects of genotoxic stress induced by γ-irradiation in mammalian oocytes are mediated by signaling pathways associated with the activation of senescence and repair from DNA damage and indicate a link between the DNA damage response and the regulation of PCH structure in the oocyte genome.

### Senescent oocytes exhibit satellite distension and extra chromosomal satellite DNA fragments

**Fig. 6:**
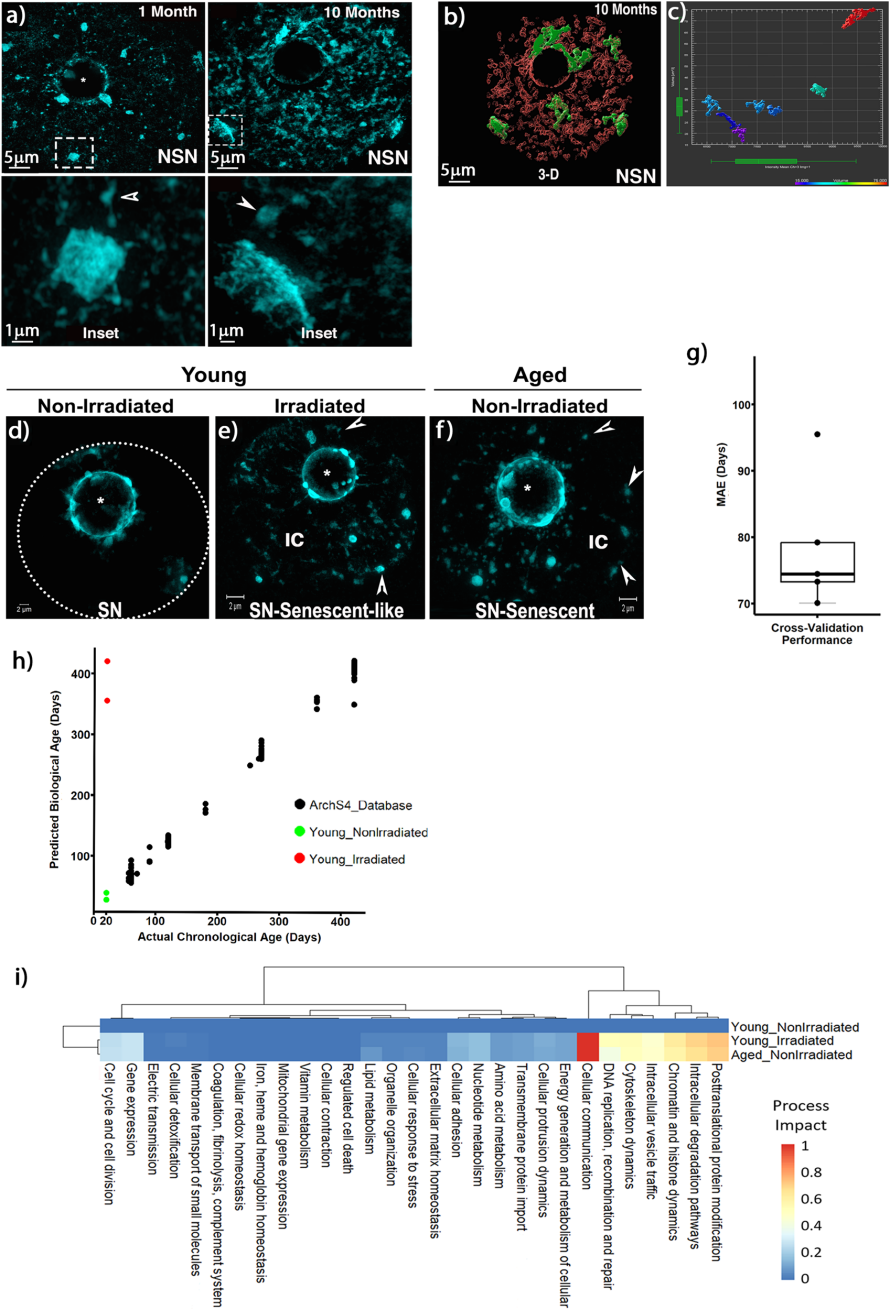
Senescent oocytes exhibit distension of major satellites and share dysregulated cellular processes with irradiated oocytes. **a** Comparison of large-scale chromatin structure between young (1-month old) and senescent oocytes (10-month old). SR-SIM resolves tightly packed chromatin fibers at condensed chromocenters in young oocytes (Inset). Senescent oocytes exhibit aberrant chromatin structure due to abnormal chromatin fiber folding and highly decondensed chromocenters (Inset). Scale bar = 5 μm. Inset scale bar = 1 μm. Arrowheads indicate euchromatin fibers. **b** 3D-SIM surface rendering of a 10-month-old oocyte showing distension of major satellite DNA fibers at chromocenters (green). Histone H2B (red). Scale bar = 5 μm. **c** Quantitative analysis of chromocenter volume (μm^3^) from the senescent oocyte shown in (**b**). **d** In young SN oocytes, chromatin is highly condensed around the nucleolus with limited contact with the nuclear periphery (dashed circle). **e** Following γ-irradiation several chromocenters detach from the nucleolus and chromatin fibers re-establish contacts with the nuclear periphery (arrowheads). Abnormal chromatin fiber folding and decondensation leads to the formation of a large interchromatin (IC) space. **f** Notably, non-irradiated senescent SN oocytes (10-month-old) exhibit a similar nuclear architecture as irradiated oocytes and chromocenters remain highly condensed. **g** Oocyte-specific RNA clock to identify cellular mechanisms dysregulated in both senescent and irradiated oocytes. Cross-validation performance of the oocyte-specific RNA clock given as mean absolute error (MAE) in days. The box plot indicates the minimum, maximum and median values and the 25% and 75% percentiles. **h** Scatter plot of actual chronological age (in days) versus predicted biological age (in days) for all training samples used. **i** Heatmap showing the level of impact on the oocyte-specific RNA clock model exerted by the dysregulation of top-level processes from the mouse version of the Molecular-Biology-of-the-Cell Ontology database.

Next, we used 3D-SIM to determine the patterns of chromatin fiber folding and quantify chromocenter volume in senescent oocytes that exhibit the NSN configuration. Young oocytes obtained from adult, sexually mature 1-month-old females exhibit highly condensed, DAPI-bright chromocenters (Fig. 6a; Inset). SR-SIM resolves tightly packed chromatin fibers at compact chromocenters (Inset). In contrast, senescent (10-month old) oocytes exhibit a highly abnormal chromatin structure. Unfolding of chromatin fibers at chromocenters induced a large-scale reorganization resulting in regions of increased fiber–fiber distance and subsequent formation of highly distended chromocenters (Fig. 6a; Inset). 3D-reconstruction of the entire nucleus revealed an increased chromocenter volume ranging between 20–75 μm^3^ in senescent oocytes (Fig. 6b, c and Supplementary Movie 10). Notably, analysis of major satellite DNA (green) and histone H2B (red) in senescent oocytes, revealed the presence of extrachromosomal (ec) satellite DNA fragments that were also labeled with H2B (Supplementary Figure 6a; arrowheads) in (7/8 = 87.5%) of senescent oocytes. Ec satellite DNA fragments were not detected in young oocytes (Fig. 2a). However, detection of chromocenter fusions and subsequent splitting in irradiated oocytes revealed the formation of highly elongated major satellite chromatin fibers (Supplementary Figure 6b; Inset and arrow). This suggests that large-scale distension of chromatin fibers contributes to the mechanism(s) of extrachromosomal DNA formation and chromosome instability.

In young, non-irradiated oocytes that exhibit the SN configuration most chromocenters are attached to the nucleolus and only a few euchromatin fibers are detected at the nuclear periphery (dashed line in Fig. 6d). However, following irradiation of young oocytes, euchromatin fibers extend to re-establish intermittent contacts with the nuclear periphery (Fig. 6e; arrowheads) leading to the formation of a prominent interchromatin space (IC), resembling the configuration observed in non-irradiated senescent oocytes (Fig. 6f) and suggesting that acute irradiation induces a senescent-like chromatin structure. Importantly, in contrast with NSN oocytes, chromocenters remain compact in both young irradiated as well as non-irradiated senescent SN oocytes. This suggests that the factor(s) involved in chromocenter compaction are lost or reduced from irradiated or senescent NSN oocytes but remain bound to chromocenters in SN oocytes, potentially reflecting distinct states of PCH epigenetic maturation. Together, our results indicate that oocyte aging is associated with increased chromocenter mobility and distension of satellite DNA sequences and suggest that chronic genotoxic stress results in the formation of extrachromosomal satellite DNA fragments and chromosome instability in senescent oocytes.

To determine conserved cellular processes that are dysregulated in both senescent and irradiated oocytes, we adapted the use of MultiTIMER, a multi-tissue RNA clock algorithm^35^ and used machine learning to develop an oocyte-specific RNA clock to establish the effects of γ-irradiation on oocyte biological aging. The model was trained using 199 transcriptome profiles of GV stage mouse oocytes of different ages using a 5-fold validation. The best fit revealed a mean absolute error (MAE) of 78.8 days in cross validation (Fig. 6g). Next, we compared the average normalized count value from two replicate transcriptomes from young non-irradiated oocytes and young irradiated oocytes from our study as well as the average normalized count value of transcriptomes from a published dataset of 14-month senescent non-irradiated oocytes^36^. Importantly, when the model was tested on the training set the mean absolute error for actual chronological age was 7.29 days and successfully predicted changes in the biological age of young, irradiated oocytes to the equivalent of >350 days (Fig. 6h; red dots). This demonstrates that our oocyte-specific RNA clock was able to identify shared transcriptomic changes associated with both cellular aging and irradiation in the training process and used these changes in the prediction. Comparison of the key cellular processes that were dysregulated in both irradiated and senescent oocytes revealed cellular communication, posttranslational protein modifications, intracellular degradation pathways as well as chromatin and histone dynamics as the top cellular processes that are associated with changes in biological aging (Fig. 6i). Our model also detected dysregulation of intracellular vesicle traffic, cytoskeleton dynamics, DNA recombination and repair, gene expression, cell cycle and cell division (Fig. 6i). These results indicate that irradiated and senescent oocytes share common cellular mechanisms of functional decline that affect genome stability and that the senescent-like chromatin structure observed in irradiated oocytes is associated with changes in biological aging induced by γ-irradiation.

## Discussion

Pericentric heterochromatin is essential for chromosome stability during female meiosis^16,17^. However, little is known about the role of satellite DNA organization in nuclear architecture. Here, we conducted a superresolution analysis of TALE-satellite DNA within its intact nuclear environment and with a level of resolution beyond the optical diffraction limit to gain mechanistic insight into the regulation of chromocenter formation and heterochromatin fiber folding in the oocyte genome. We provide evidence for a dynamic spatial rearrangement of major satellite DNA suggesting that large-scale movements and interactions between different homologous chromosome bivalents contribute to chromocenter formation in the germinal vesicle. Our results suggest the presence of a previously unrecognized developmental transition in the number and volume of chromocenters in preparation for meiosis onset. Transcriptionally inactive SN oocytes exhibit remarkably mobile chromocenters that may fuse or split while dynamically interacting with the nucleolus, and γ-irradiation further enhanced chromocenter mobility. In contrast, transcriptionally active (NSN) oocytes exhibit unfolding of chromatin fibers, and a striking distension of satellite DNA following irradiation. This process is associated with enhanced expression of factors required for protection from oxidative stress, recovery from DNA damage and regulation of heterochromatin assembly. Notably, non-irradiated senescent oocytes exhibit inherently high chromocenter mobility and distension of satellite DNA. Together, our results indicate that increased chromocenter mobility and abnormal chromatin fiber folding at PCH result in the formation of extrachromosomal DNA fragments in senescent oocytes and contribute to the mechanisms of chromosome instability during oocyte aging.

Repetitive DNA constitutes >40% of the mammalian genome and is critical for chromatin organization^37^. The number and distribution of chromocenters are cell-type specific and reflect the epigenetic state acquired during differentiation^37,38^. In somatic cells, major satellites from several chromosomes associate to form a chromocenter. Notably, the centromeres of each chromosome remain as two separate foci that can be clearly resolved by SR-SIM^18,39^. In contrast, superresolution analysis of both minor and major satellites in oocytes revealed the presence of fused centromeres on each homologous chromosome bivalent and suggests that large chromocenters are formed by the coalescence of major satellites from several homologous chromosome bivalents. Thus, by bringing multiple homologous chromosomes together at each chromocenter, PCH may directly affect the formation and stability of long-range chromosome interactions in the oocyte genome. The mechanisms regulating the association of major satellite DNA between different chromosomes are not known. Major satellite clustering may be mediated by heterochromatin binding proteins, epigenetic modifications, and potentially non-coding RNAs^40,41^. However, the specific factors that mediate the coalescence of satellite DNA into chromocenters remain to be determined. Notably, an increase in chromocenter number and a concomitant reduction in their volume in SN oocytes suggest the presence of a developmental transition whereby chromocenters become reorganized in preparation for meiotic cell division to ensure accurate chromosome segregation.

Partial 3-D reconstruction using electron spectroscopic imaging in mouse fibroblasts revealed that chromocenter formation involves an increase in chromatin fiber density and compact chromatin fiber folding^42^. Consistent with that study, SR-SIM resolves highly compact heterochromatin fibers in the oocyte chromocenters. Importantly, in contrast with electron spectroscopic imaging tomography, SR-SIM is compatible with protein localization and resolved the association of ATRX with individual major satellite DNA fibers. Loss of ATRX function is associated with centromere instability during oocyte meiosis^16,43^ consistent with a role in the regulation of heterochromatin structure. However, the specific factors regulating chromatin fiber folding and fiber–fiber density in the oocyte chromocenters remain to be determined.

In somatic cells, the spatial organization of chromocenters is regulated by interactions with the nucleolus^40^. Our results indicate that oocyte chromocenters exhibit high mobility and establish dynamic interactions with the nucleolar surface. In SN oocytes, some chromocenters exhibited a combination of linear and non-linear trajectories with a total path length of up to 60–70 μm, suggesting remarkable chromocenter dynamics compared with somatic cells. For example, stochastic analysis of relative trajectories indicates that in human cells, centromeres exhibit constrained relative motion within a few microns^44^. In HeLa cells, centromeres have been shown to remain mostly stationary but can occasionally move at speeds up to 7–10 μm/hour^45^. Importantly, it has been suggested that short range, constrained centromere motion is critical to prevent the loss of chromosome folding^44^. Our results provide evidence indicating that oocyte chromocenters fuse and split and that dynamic interactions between PCH domains of different chromosomes also take place at the nucleolar surface. Thus, regulation of heterochromatin dynamics is of direct consequence for genome integrity^44,46^.

Acute γ-irradiation significantly increased chromocenter mobility in SN oocytes due to detachment from the nucleolus and changes in large-scale chromatin organization. Within 30 min, the patterns of chromocenter oscillatory movements changed into a linear displacement, resulting in a significant increase in the average chromocenter speed and total path length suggesting that genotoxic stress enhanced global chromatin mobility. In somatic cells, changes in chromatin motion are an integral component of the cellular response to DNA damage and specific genomic loci exhibit increased mobility over long distances in order to facilitate the repair of double-strand breaks^47–52^. Our results indicate that DNA damage can also induce large-scale movements of heterochromatin domains in the oocyte genome.

In human and mouse somatic cells, SADs formation is a marker of early senescence associated with changes in large-scale chromatin organization^4,53^. However, the mechanisms of SADs formation are not fully understood. Our results indicate that γ-irradiation induced distension of major satellites within 24 h in transcriptionally active (NSN) oocytes due to large-scale unfolding of chromatin fibers. ATRX is known to interact with HP1 in heterochromatin formation and is critical for centromere stability in mouse oocytes^16,19,54,55^. However, ATRX remained associated with PCH fibers in irradiated oocytes suggesting that unfolding of major satellite DNA takes place in spite of ATRX localization. Consistent with this observation, a recent study indicates that neither the size, nor the state of chromatin compaction at chromocenters is dependent on the levels of HP1 in mouse fibroblasts^41^. Notably, Fortuny et al. recently demonstrated a significant reduction in PCH compaction following targeted UV damage^56^. However, several repressive heterochromatin marks persist at decondensed chromocenters suggesting that retention of remodeling factors may be part of a maintenance mechanism to safeguard heterochromatin organization and facilitate recovery from DNA damage^56^.

Chromocenters are formed by groups of chromatin fibers that organize into a densely packed and highly compact structure^42,53^. In mouse fibroblasts, chromocenters resemble collapsed polymer globules that can adopt condensed or decondensed folding states reflecting important mechanisms of chromatin organization^41^. The factors responsible for regulating chromatin fiber folding and fiber–fiber cross-links to maintain high-density DNA packing remain to be established. However, transcriptome analysis of irradiated oocytes indicates that unfolding of major satellite DNA fibers is associated with changes in factors required for regulation of heterochromatin formation. Transcripts for the zinc finger protein *Zfp296* exhibited a 6-fold overexpression. Zfp296 is localized to pericentric heterochromatin in mouse post-implantation embryos where it acts as a negative regulator of H3K9me3^21^. However, γ-irradiation also induced a 20-fold overexpression of transcripts for the *Ski proto-oncogene*. Ski localizes to PCH and is required to maintain the levels of H3K9me3 and transcriptional repression of several genes (*Mmp3, Mmp10, Mmp13*) located within major satellites of chromosome 9^24^. Interestingly, Ski overexpression induced desensitization of human epithelial cells to genotoxic drugs^57^ suggesting a potential role in protection from DNA damage. Our results indicate that γ-irradiation may disrupt the balance between expression of positive and negative regulators of histone modifications and transcriptional silencing of PCH domains thus interfering with the establishment of critical epigenetic marks required for chromocenter compaction in the oocyte genome.

The actin-related kinase (Actr3) maintains chromatin organization and influences chromatin dynamics through its role in the regulation of nuclear lamina integrity^22^. Transcripts for *Actr3* were overexpressed >10 fold following oocyte irradiation, potentially contributing to changes in large-scale chromatin organization and suggesting that changes in chromocenter dynamics may be mediated by abnormal interactions with the nuclear lamina. Importantly, our results revealed that coincident with changes in mesoscale chromatin organization after irradiation, the oocyte also triggers an active transcriptional response to DNA damage. Consistent with this notion, we detected a significant overexpression of factors required for protection from oxidative stress (*Fermt1, Smg1*), restoration of chromatin structure at DNA breaks (*Tlk2*), DNA repair and maintenance of genome stability (*Rad54l*). The human FERMT-1 gene encodes for Kindlin-1^25^. Kindlin-1 plays a key role in regulating the cellular response to acute oxidative stress^25^. Notably, it is required for mitotic spindle assembly^58^. Smg1 functions in the response to genotoxic stress, DNA damage, and maintenance of telomere integrity^26^. The Tousled-like kinase 2 (Tlk2) is critical for recovery following DNA damage at the G2 phase where it regulates the histone chaperone Asf1A to restore histone H3 deposition into damaged chromatin^27^. The DNA translocase RAD54l promotes accurate repair of DNA damage to prevent chromosome segregation defects^28^. Notably, γ-irradiation also induced transcriptional changes associated with the activation of cellular senescence and regulation of chromatin structure (*Ctcf, Napl4, Rev1, Dnmt3L*). Amongst the most significantly downregulated factors, we detected a >20-fold reduction in transcripts for *Cingulin*, a tight junction protein localized to the sites of contact between cumulus cells and the oocyte cytocortex^59^. In Zebra fish embryos, loss of Cingulin activates cellular senescence pathways^29^. Transcripts for *Rev1* were downregulated >5 fold. Notably, inhibition of Rev1 is associated with activation of senescence in human ovarian cancer cell lines and mouse fibroblasts^60^. Transcripts for *Birc6/Bruce* were downregulated >20 fold. Birc6 is required for homologous recombination in somatic cells and prevents non-homologous chromosome interactions during spermatogenesis^32^. Collectively, our results indicate that γ-irradiation activates signaling pathways associated with the onset of cellular senescence as well as repair from DNA damage and suggest a mechanistic link between the DNA damage response and regulation of large-scale heterochromatin structure in the oocyte genome. Studies are in progress to identify the specific protein factor(s) or potential histone post-translational modifications that induce the loss of chromocenter compaction in NSN oocytes.

We provide evidence indicating that non-irradiated senescent oocytes with the SN configuration exhibit similar chromocenter mobility compared to irradiated oocytes. Notably, senescent oocytes that remained at the NSN configuration also exhibited distension of major satellite sequences. This suggests that acute genotoxic stress following γ-irradiation and chronic genotoxic stress resulting from cellular aging induce major satellite distension. Comparison of the cellular mechanisms that are dysregulated in both senescent and irradiated oocytes using an RNA clock revealed that abnormal cellular communication, chromatin dynamics, and histone posttranslational modifications are important mechanisms associated with the formation of abnormal chromatin structure in both senescent and irradiated oocytes and resulted in the advanced biological age detected in irradiated oocytes. Previous studies suggested that SADs formation in human senescent cells may be due to abnormal chromatin folding^4^. More recently, STED microscopy revealed that SADs are formed by globular domains linked by threads of distended DNA^53^. Our direct visualization of major satellite chromatin fibers in mouse oocytes revealed that changes in chromocenter compaction are due to both, unfolding of chromatin fibers and increased fiber–fiber distance that result in a significant increase in chromocenter volume.

A previous study using diffraction limited imaging detected an indiscernible chromatin configuration in aged mouse oocytes^61^. Our SR-SIM analysis provides evidence that NSN senescent mouse oocytes exhibit distension of major satellites, abnormal chromatin fiber folding and disrupted large-scale chromatin organization. To the best of our knowledge, this is the first report of abnormal chromocenter formation during in vivo oocyte senescence. Our results are consistent with the notion that major satellite distension is due to changes in higher-order packaging of heterochromatin. The factor(s) responsible for maintaining the highly compact state of chromocenters and packing density of satellite DNA fibers remain to be established. However, the differential response to both γ-irradiation and senescence observed between NSN and SN oocytes provides intriguing new insight. Indeed, in irradiated SN oocytes chromocenters remain highly compact, albeit with increased mobility suggesting that the factor(s) responsible for regulating major satellite fiber packing and chromocenter condensation are probably lost from irradiated or senescent NSN oocytes but remain bound to chromocenters in SN oocytes. Thus, it is conceivable that this may reflect a previously unappreciated step in the epigenetic maturation of heterochromatin domains in pre-ovulatory oocytes. The effect(s) of SADs formation on centromere structural integrity and chromosome segregation are not fully understood. However, our recent studies in senescent mouse fibroblasts indicate that SADs formation is associated with centromeric breaks and severe chromosome instability^3^. Consistent with this notion, a high proportion of senescent oocytes exhibit extrachromosomal satellite DNA fragments, a sign of chromosome instability. Notably, the co-localization of H2B and extrachromosomal major satellite DNA indicates that these fragments are still packed within nucleosome-containing chromatin. Previous studies indicate that precocious bivalent separation may be the primary defect in age-related aneuploidy^62^. However, the mechanisms involved in this process remained unclear. Our studies provide evidence indicating that abnormal pericentric heterochromatin structure, increased chromocenter mobility and distension of satellite DNA contribute to the formation of extrachromosomal satellite DNA and chromosome instability, predisposing to abnormal chromosome segregation in senescent oocytes. Our findings have implications not only for the field of onco-fertility but also to determine the transcriptional and epigenetic mechanisms of oocyte senescence and genome instability.

## Methods

### Animals

Animal use protocols were approved by the ‘Institutional Animal Care and Use Committee’ (IACUC) of the University of Georgia, and all experiments were conducted in accordance with guidelines. Mice were housed at a constant temperature (24–26 °C) and a controlled light cycle (12 h light/dark), with food and water provided ad libitum.

### Oocyte collection and culture

All experiments involved GV stage oocytes collected from non-primed female mice with a C57/Bl6/DBA2J F1 hybrid background between postnatal days 16 to 24 as well as from aged females (10 months). The oocytes were recovered from the ovaries by follicle puncture and cumulus oocyte complexes were transferred to minimal essential medium (MEM) supplemented with 3 mg/ml bovine serum albumin (Sigma Aldrich, St. Louis, MO) and milrinone (Sigma; 1 μg/ml) to prevent meiotic resumption. Surrounding cumulus cells were removed by gentle pipetting followed by several rinses in fresh medium. Oocytes were cultured for specified periods of time and maintained at 37 °C in MEM/BSA plus milrinone under 5% CO_2_, 5%O_2_, and balanced N_2_.

### Immunofluorescence

Denuded oocytes were fixed at indicated timepoints post irradiation and permeabilized in 2% paraformaldehyde in PBS supplemented with 0.1% Triton X-100 for 20 min at room temperature before washing and blocking in PBS supplemented with 5% serum and 0.05% Triton X-100 overnight at 4 °C. Oocytes were immunolabeled with specific antibodies against the DNA double-strand break marker γH2AX (1:400, cat# ab26350, Abcam, Cambridge, USA), and the heterochromatin marker ATRX (1:200, cat# sc15408, Santa Cruz Biotechnology Inc., Dallas, TX). In brief, fixed oocytes were incubated with the primary antibodies diluted in blocking buffer overnight at 4 °C, then washed and incubated with specific Alexa Fluor 488 or 555 conjugated secondary antibodies (1:1000, Cat# A11017, A21430, Life Technologies, Eugene, OR) for 1 h at 37 °C. After a series of final wash steps in blocking buffer, the oocytes were transferred onto glass slides and overlaid with mounting medium (VectaShield, Vector Laboratories, Burlingame, CA) containing DAPI (4’,6-diamidino-2-phenylindole) to counterstain the DNA.

### Oocyte microinjection

We analyzed chromatin dynamics in live oocytes, using recombinant Histone H2B-RFP and a TALEN vector specific for centromeric major satellite sequences. Briefly, GV stage oocytes were microinjected with capped mRNAs encoding fluorescently labeled histone H2B-fusion proteins (H2B-RFP, red) as well as transcription activator-like effector (TALE)-mClover transcripts against repetitive major satellite genomic sequences (green) following vitro transcription from plasmids pGEMHE-H2B-RFP (Euroscarf, cat# P30517)^63^ and pTALYM3B15 (Addgene cat# 47878)^14,19^. In brief, 10 pl capped mRNA were microinjected into the cytoplasm of GV-intact oocytes in MEM medium containing 1 μg/ml milrinone using an electronic FemtoJet microinjector (Eppendorf) and Eppendorf micromanipulators on a Nikon Eclipse Ti-U inverted microscope. Oocytes were incubated overnight to allow for the expression of the fluorescent labels.

### Oocyte irradiation

For the analysis of the effects of γ-irradiation, oocytes were washed and transferred to a micro drop of Embryomax M2 medium (Sigma, cat# MR-015-D) supplemented with milrinone under mineral oil and subjected to a non-lethal dose of γ-irradiation (5 Gy; 140 Rad/min for 3.57 min) using a Mark I 68A Cesium-137 gamma irradiator (JL Shepherd and Associates, San Fernando, CA). Oocytes were then transferred to a fresh micro drop of MEM/BSA medium supplemented with milrinone under mineral oil and cultured for 30 min, 5 h, or 24 h before fixing and immunochemistry as described above. Alternatively, chromatin domain movements were monitored in real time by live-cell imaging and confocal microscopy, followed by SR-SIM analysis as described below.

### Live-cell-imaging

Live-cell-imaging was conducted by confocal microscopy on groups of irradiated and non-irradiated oocytes in micro drops of MEM/BSA medium supplemented with milrinone under mineral oil in an environmental chamber with an atmosphere of 5% CO_2_, 5% O_2_, and 90% N_2_. Oocytes were imaged using an Eclipse Ti-U/D-Eclipse C1 laser-scanning confocal microscope equipped with a ×40 objective lens following sequential (frame lambda) excitation of mClover/EGFP with a 488 nm and H2B-RFP with a 561 nm Coherent Sapphire laser. Image acquisition was conducted using EZC1 3.91 software (Nikon) with a step size of 2 μm and a Z-stack range of ~60 μm. Images were acquired every 5 min beginning at 30 min post irradiation for 5 h and subsequently analyzed by maximum intensity and 3D-reconstructions using NIH Elements 4.0 software and IMARIS (Bitplane, Inc.). Heterochromatin domains labeled with TALE-mClover were tracked in 3D using the tracking module in NIS Elements applying identical thresholds on a Nikon Eclipse Ti-U/D-Eclipse C1 laser scanning confocal microscope equipped with a ×40 objective. Speed in µm/s and the total path length in µm were assessed for each chromocenter individually. Image acquisition was conducted using EZ-C1 software (Nikon) with a step size of 2 µm and a *Z*-stack range of 20 µm. Imaging data were subsequently analyzed by maximum intensity and 3D-reconstructions using NIH Elements software (Nikon).

### Superresolution structured illumination

Oocyte chromatin was analyzed by high-resolution structured illumination microscopy (SR-SIM)^15^. Imaging was conducted using a Zeiss ELYRA S1 Superresolution system on an Axio Observer Z1 inverted microscope stand equipped with a 100X oil immersion lens and three laser lines: 405, 488, and 561 nm. Images were generated using 3 SIM rotations and individual Z-scans (0.091 μm) were processed with ZEN 2.3 software with a SIM analysis module to generate SR-SIM computations and maximum intensity projections at the Biomedical Microscopy Core (BMC) facility, University of Georgia. Quantitative analysis of chromocenter organization was conducted using Imaris x64 9.5.0 software (Bitplane). CZi files containing maximum intensity projections of the entire germinal vesicle were imported into Imaris. Pericentric heterochromatin blocks (chromocenters) were initially classified based on the DAPI fluorescence and major Satellite TALE-mClover channels using the automatic new surface function. Surface grain size was set at 0.0500 μm. The thresholds were 8700 for the absolute DAPI intensity and 2.30 μm^3^ for the volume so that DAPI intense heterochromatin blocks assembled as chromocenters could be automatically classified. 3D-surface-renderings of individual chromocenters as well as select chromatin fibers were automatically generated. We used the manual ‘wizard’ tool to draw the surface of the entire nucleus or germinal vesicle and the animation function of Imaris to generate a 3D-rendering rotating 720 degrees in the horizontal direction. The Vantage tab was used for quantitative analysis of chromocenter volume where the Y-axis displays chromocenter volume in μm^3^ and the X-axis indicates mean DAPI intensity. All epifluorescence images were processed using Photoshop 2.0 (Adobe) for linear adjustments and cropping of fluorescent images. No gamma adjustments were made.

### Transcriptome analysis following γ-irradiation of NSN oocytes

Transcriptionally active NSN oocytes obtained on day 16 of post-natal development were processed for RNA-seq by first removing the zona pellucida with Tyrode’s solution (Sigma) and allowed to recover in MEM/BSA culture medium for 5 min. Control (*n* = 59) and γ-irradiated (*n* = 59) denuded oocytes were transferred to microcentrifuge tubes containing 3 μl of lysis buffer (40 U/ml RNase Inhibitor, 10% Triton X-100, 10 mM dNTPs in nuclease-free water) and immediately frozen in liquid nitrogen. Analysis was conducted in two independent experimental replicates. Total RNA quality on each sample was determined using the Agilent 2100 Bioanalyzer system. Quality control prior to library construction included RNA concentration, an RNA integrity number of >8, 28/S/18S of >1.0 and fragment length distribution. Isolation of mRNA was conducted using oligo(dT). Library sequencing was performed using a Hiseq 4000 sequencing platform with paired-end 100 bp reads at the Beijing Genomics Institute. For each sample, >88 Million clean PE 100 reads or 8.93 Gb of data were obtained in FASTQ format. Quality-trimmed reads were aligned to the mouse genome reference GRCm38 using Bowtie2. Differential expression analysis between control and irradiated oocytes was conducted using the edgeR package. Transcripts with a log2-fold change of >2 and FDR < 0.05 were considered significant for differential expression (DEGs). DEGs were detected using DEseq2. For functional annotation, the gene ontology functional enrichment analysis and the Kyoto Encyplopedia of Genes and Genomes (KEGG) were used for DEGs classification using the Database for Annotation, Visualization and Integrated Discovery (DAVID). The data discussed in this publication have been deposited in NCBI’s Gene Expression Omnibus and are accessible through GEO Series accession number GSE248416.

### Validation of RNAseq by qPCR

For qPCR assays to validate differential expression following irradiation, mRNA was isolated from groups (*n* = 50) of denuded oocytes control and irradiated (24 h) oocytes using the miRNeasy kit (Qiagen) and subsequently reverse transcribed according to manufacturer’s instructions to assess transcript levels using validated RT^2^ qPCR Primer Assays (Qiagen; GeneGlobe IDs: Rev1-QT00150437, Dnmt3l-QT00114555, TLK2-QT00136010, Rad54L-QT00104132) in two biological replicates. qPCR results were normalized against Gapdh (RT^2^ qPCR Primer assay, Qiagen; GeneGlobe ID: Gapdh-PPM02946E) as a housekeeping control.

### Analysis of biological (cellular) aging following γ-irradiation using an oocyte-specific RNA clock

We adapted the use of a multi-tissue RNA clock MultiTIMER^35^ in combination with machine learning to identify the common pattern of transcriptomic changes induced by cellular aging and irradiation in mouse oocytes. The generalized linear model (GLM) algorithm of MultiTIMER^35^ was replaced by the gradient boosting machine (GBM) algorithm^64^, and the model was trained on 199 transcriptome profiles from the ArchS4 database of GV, control wild-type oocytes from mice of different ages. We also added the two control and two irradiated transcriptomes from our database into the training. To discover conserved cellular processes that are dysregulated in both aged and irradiated oocytes, the model was trained to predict the age of transcriptome profiles of non-irradiated oocytes accurately while also predicting advanced biological age for the transcriptome of irradiated oocytes. The h2o framework in R was used for machine learning applications^65^.

To identify the most important cellular processes affected in aged and irradiated oocytes we compared the average normalized count value from two replicate transcriptomes from young non-irradiated oocytes and young irradiated oocytes from our study as well as the average normalized count value of transcriptomes from a published dataset of 14-month aged non-irradiated oocytes^36^. These datasets all participated in the training of the model.

We established our young control transcriptome as the baseline transcriptome. Then, in both young-irradiated and aged non-irradiated transcriptomes, we calculated the gene impact of all genes in the 29 top-level processes in the mouse version of the Molecular-Biology-of-the-Cell Ontology (process level 1)^66^. We define gene impact as the influence of a transcriptomic change of a gene in model prediction, and it is calculated by:

$${{{{{{\rm{Gene}}}}}}\; {{{{{\rm{impact}}}}}}}=I* {{{{{\rm{|}}}}}}D{{{{{\rm{|}}}}}}$$

where *I* is the variable importance of the gene in model and *D* is the difference in normalized count value from baseline.

The sum of gene impact in each process is defined as the process impact, which indicates the level of impact on the model prediction exerted by the dysregulation of these conserved cellular processes. These process impact values were normalized to a range of 0–1 and drawn on a heatmap.

### Statistics and reproducibility

All data are presented as the mean values (±s.d.) from at least three independent experimental replicates unless stated otherwise. Comparison of all pairs was conducted using two-sided parametric and nonparametric tests (unpaired t-test or Mann–Whitney tests) according to the sample distribution (D’Agostino-Pearson) with GraphPad Prism 6 software or ggplot2 framework in R. Analysis of variance was conducted using one-way ANOVA with Tukey-s multiple comparison tests. Differences were considered significant when *P* < 0.05 and *P* values are indicated. Box plots depict the median value as well as upper and lower quartiles with whiskers representing the minimum and maximum values observed. Dot plots show individual observations as dots as well as the mean with whiskers representing the standard deviation (s.d.).

### Reporting summary

Further information on research design is available in the Nature Portfolio Reporting Summary linked to this article.

### Supplementary information

Supplementary Information

Description of Additional Supplementary Files

Supplementary Data 1

Supplementary Movie 1

Supplementary Movie 2

Supplementary Movie 3

Supplementary Movie 4

Supplementary Movie 5

Supplementary Movie 6

Supplementary Movie 7

Supplementary Movie 8

Supplementary Movie 9

Supplementary Movie 10

Reporting Summary

## Data Availability

The data discussed in this publication have been deposited in NCBI’s Gene Expression Omnibus and are accessible through GEO Series accession number GSE248416. The source data behind the graphs in the paper can be found in Supplementary Data 1. All other data are available from the corresponding author on reasonable request.
