# Supplementary material for: Acute irradiation induces a senescence-like chromatin structure in mammalian oocytes
Source: Commun Biol. 2023 Dec 12;6:1258. doi: 10.1038/s42003-023-05641-0 (PMC10716162; doi:10.1038/s42003-023-05641-0)
Supplement: Supplementary file 1 — Supplementary Information [file 42003_2023_5641_MOESM1_ESM.pdf]

Supplementary Information

Communications Biology

Baumann et al. 2023

Acute irradiation induces a senescence-like chromatin structure in mammalian oocytes

Supplementary Figure 1

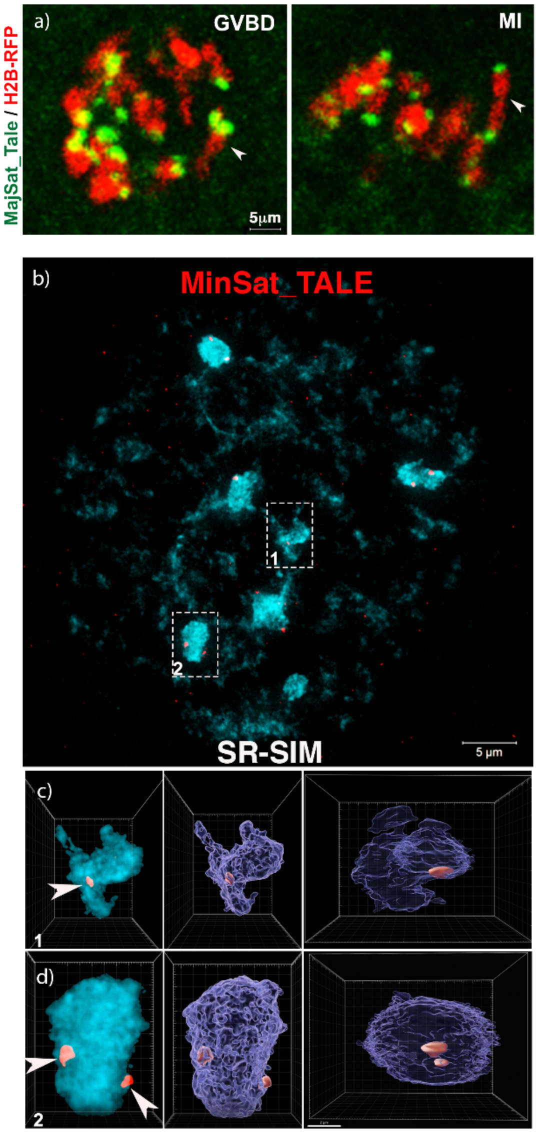

Supplementary Figure 1. Detection of centromere minor and major satellite DNA sequences using live cell imaging and superresolution structured illumination (SR-SIM). (a) Live oocytes microinjected with a transcriptional activator-like effector (majSat_TALE, green) and Histone H2B-RFP (red). Major satellite_TALE reveals the physical proximity of pericentric heterochromatin domains in a homologous chromosome bivalent after germinal vesicle breakdown (GVBD) and their polar orientation at the metaphase-I (MI) stage. Uncropped micrographs of images in Figure 1B. **(b)** Germinal vesicle stage oocyte microinjected with a MinSat_TALE effector (red). Chromatin was counterstained with DAPI (shown in cyan) revealing the presence of chromocenters of varying sizes (dashed boxes 1 and 2). Scale bar=5 μm. (**c)** Inset dashed box 1. The presence of a single minor satellite signal (arrowhead) indicates that this chromocenter is formed by a single homologous chromosome bivalent. 3-Dimentional surface rendering of the same chromocenter showing the presence of fused centromeres (red). Lateral view. **(d)** Inset dashed box 2. Two minor satellite signals (red) in opposite sides of large chromocenters (arrowheads) confirm the presence of fused centromeres, with each fused centromere signal corresponding to a single homologous chromosome bivalent. 3-Dimentional surface renderings of the same chromocenter showing the localization of fused centromeres (red). Scale bar=2 μm.

Supplementary Figure 2

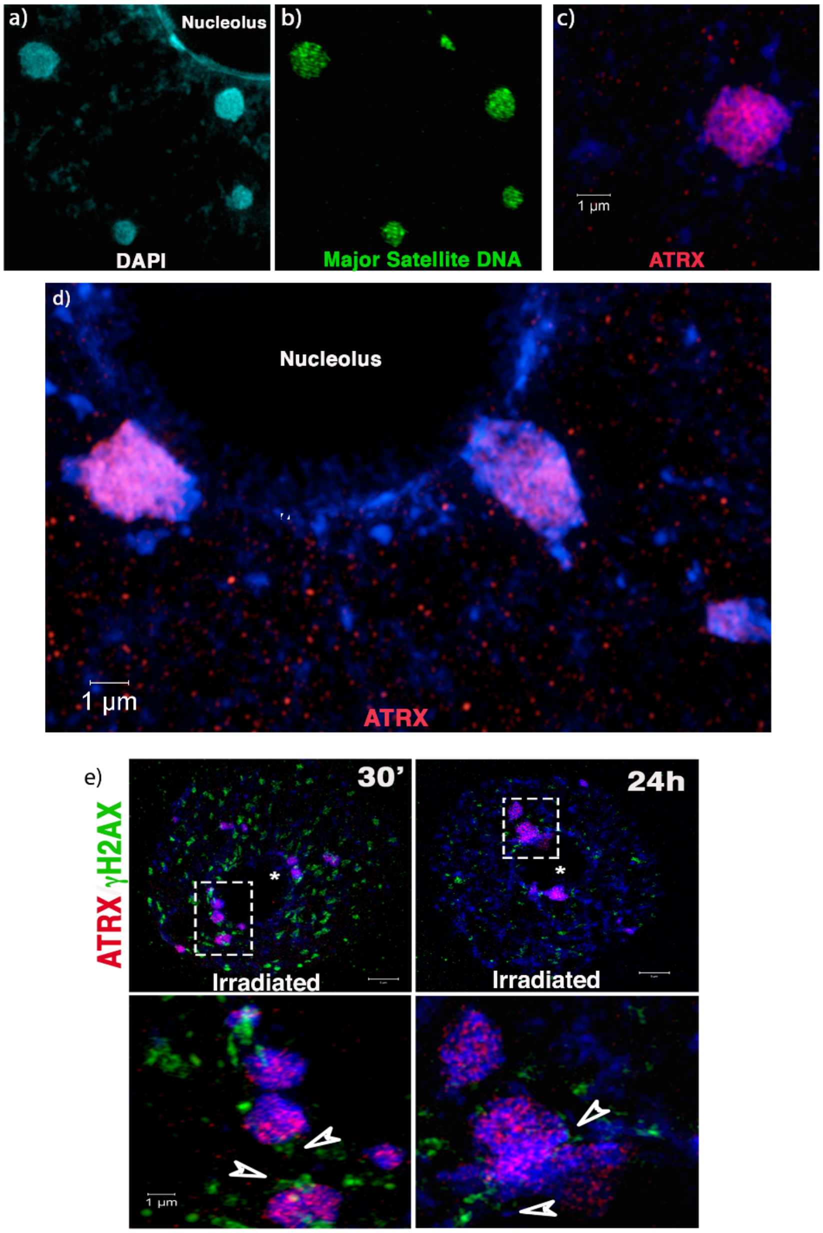

Supplementary Figure 2. ATRX localization at oocyte chromocenters. a) Superresolution Structured illumination (100X) of DAPI bright chromocenters. **b)** Corresponding major satellite chromatin fibers (green). **c)** ATRX (red) decorates a subset of intertwined major satellite chromatin fibers within compact chromocenters in a different NSN oocyte. DNA counterstained with DAPI. **d)** Chromocenters begin to associate with the nucleolus in the same oocyte. ATRX (red). Scale bar=1 µm. **e)** γ-irradiation (5 Gy) induces double strand DNA breaks, detected by γ-H2AX (green) within 30 minutes after irradiation. However, ATRX (red) remains associated with compact chromocenters. In contrast, 24 hours following irradiation of NSN oocytes, chromocenters undergo a striking decondensation while ATRX remains associated with major satellite chromatin fibers.

Supplementary Figure 3

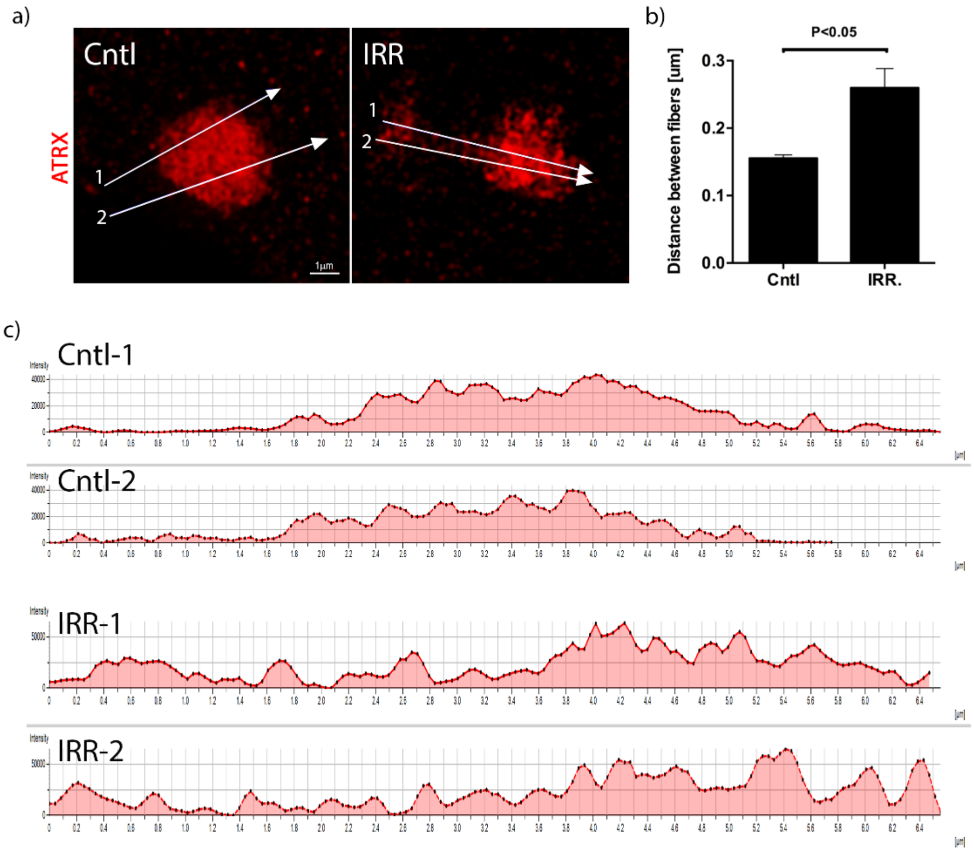

Supplementary Figure 3. Distension of major satellite DNA following γ-irradiation. a) Superresolution structural illumination (100X) resolves unfolding of heterochromatin fibers stained with ATRX (red) and subsequent increase in fiber-fiber distance in chromocenters of NSN oocytes 24h post irradiation. **(b, c)** Quantification of fiber-fiber distance (n=46 fibers) using line-scan analyses in the NIS Elements measurements module. Irradiation induces a significant increase ($P<0.05$) in fiber-fiber distance of satellite DNA compared to non-irradiated control oocytes. Scale bar=1 μm.

Supplementary Figure 4

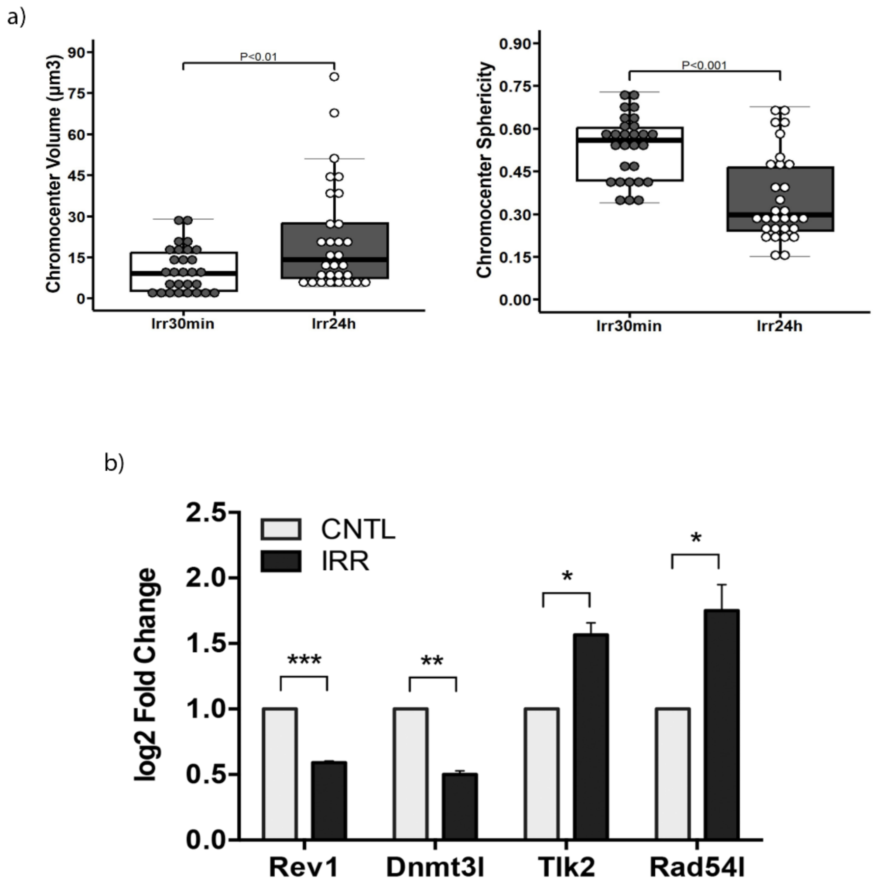

Supplementary Figure 4. Analysis of chromocenter volume and validation of transcriptional changes following γ–irradiation of NSN oocytes. a) Analysis of chromocenter volume and sphericity at different time intervals following γ-irradiation of NSN oocytes. **b)** Real-time PCR analysis of transcripts showing down regulation or over expression after γ-irradiation of pools of control (n=50) and irradiated (n=50) oocytes. NSN oocytes were obtained on day 16 of post-natal development and transcripts levels were quantified 24 h following γ-irradiation (5 Gy) in two independent experimental replicates. * indicates P<0.05; ** P<0.01; *** P<0.005.

Supplementary Figure 5

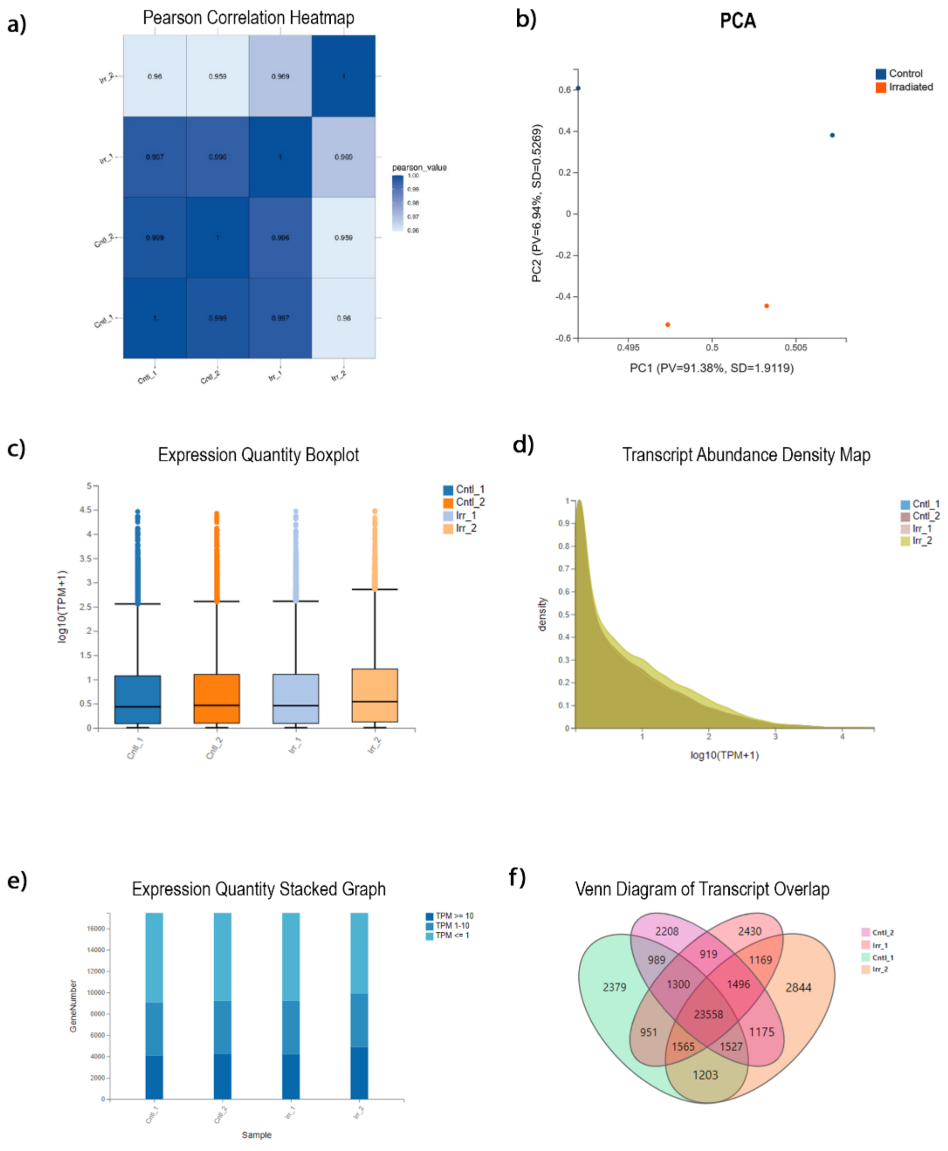

Supplementary Figure 5. Transcriptome analysis quality control. a) Pearson correlation analysis between control and irradiated RNA-seq samples. **b)** Principal component analysis showing segregation of samples by treatment. **c)** Box plot of the distribution of gene expression levels between samples. **d)** Transcript abundance density distribution between samples. **e)** Number of genes by different TPM ranges across all samples. **f)** Venn diagram of transcript overlap between samples.

Supplementary Figure 6

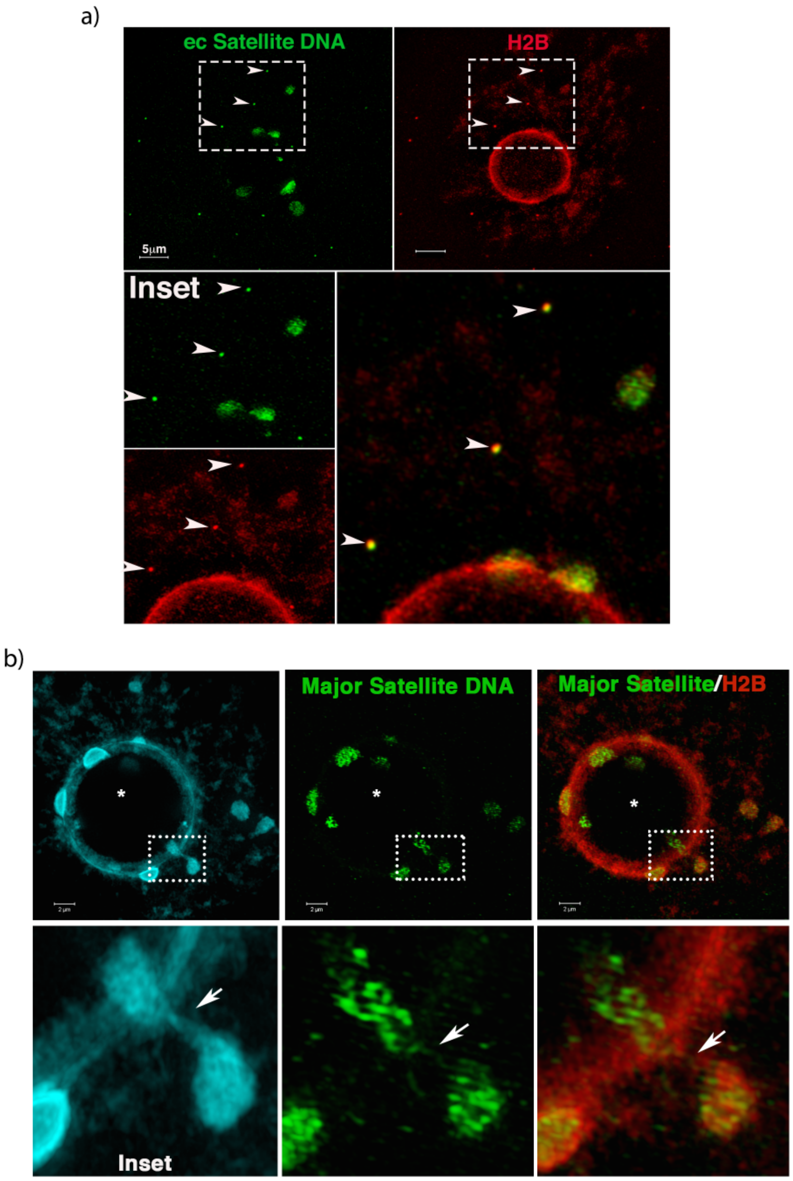

Supplementary Figure 6. Evidence for the mechanism(s) of formation of extrachromosomal satellite DNA in the oocyte genome. a) Detection of extrachromosomal satellite DNA fragments in senescent oocytes. A representative 10-month-old oocyte is shown. Fragments of major satellite DNA (arrowheads) also stain positive for histone H2B (red). See Insets. Scale bar=5 µm. **b)** Chromocenter fusions and subsequent splitting in irradiated oocytes leads to excessive distention of major satellite chromatin fibers (arrows at insets) revealing a potential mechanism of extra chromosomal satellite DNA formation in the oocyte genome. (*) Demarcates the nucleolus.
