# Supplementary material for: Acute irradiation induces a senescence-like chromatin structure in mammalian oocytes
Source: Commun Biol. 2023 Dec 12;6:1258. doi: 10.1038/s42003-023-05641-0 (PMC10716162; doi:10.1038/s42003-023-05641-0)
Supplement: Supplementary file 2 — Description of Additional Supplementary Files [file 42003_2023_5641_MOESM2_ESM.pdf]

Description of Additional Supplementary Files

File name: Supplementary Data 1
Description: The source data behind the graphs in the paper.

File name: Supplementary Movie 1
Description: Live-cell-imaging of chromocenter mobility in non-surrounded nucleolus (NSN) oocytes. Composite image of confocal microscopy (40X) following microinjection of a fluorescent TALE effector labeling major satellite DNA (green) and simultaneous microinjection of recombinant RFP-H2B (red). Individual chromocenter trajectories were tracked for a period of 5 hours. Large chromocenters exhibit oscillatory movements. Small chromocenters also exhibit dynamic interactions and fusions (white arrow) with major satellite sequences from adjacent homologous chromosome bivalents.

File name: Supplementary Movie 2
Description: Live-cell-imaging of chromocenter mobility in surrounded nucleolus (SN) oocytes. Composite image of confocal microscopy (40X) following microinjection of a fluorescent TALE effector labeling major satellite DNA (green) and nuclear counterstaining with recombinant RFP-H2B (red). Individual chromocenter trajectories were tracked for a period of 5 hours. Most chromocenters are attached to the nucleolus and exhibit dynamic interactions leading to fusion and splitting events. A few chromocenters remain unattached and show high mobility.

File name: Supplementary Movie 3
Description: 3-Dimensional surface rendering of a large chromocenter with two minor satellite signals. Superresolution structured illumination (100X) reveals the presence of fused centromeres (red) on opposite sides of a large chromocenter indicating that in NSN oocytes, large chromocenters are formed by the interaction and coalescence of two homologous chromosome bivalents. Fused centromeres are localized towards the periphery indicating that minor satellite sequences at the centromere remain in close apposition albeit spatially segregated from pericentric heterochromatin major satellite sequences that form the chromocenter.

File name: Supplementary Movie 4
Description: 3-Dimensional surface rendering of a chromocenter formed by a single bivalent. Superresolution structured illumination (100X) reveals the presence of only one minor satellite signal (red) representing the fused centromeres of a single bivalent.

File name: Supplementary Movie 5
Description: Superresolution structured illumination (3D-SIM) of the oocyte nucleolus. 3D-Rendering of the nucleolus (100X) from an oocyte that exhibited the SN configuration. DNA stained with DAPI and pseudo colored in Cyan for contrast. 3D-SIM resolves compact chromatin

fibers at pericentric heterochromatin as well as topological differences at individual chromocenters that are attached to the nucleolus. Note the splitting of large-scale chromatin fibers within an attached chromocenter.

File name: Supplementary Movie 6
Description: Live-cell-imaging of chromocenter mobility in control non-irradiated SN oocytes. Composite image of confocal microscopy (40X) following microinjection of a fluorescent TALE effector labeling major satellite DNA (green) and recombinant RFP-H2B (red). Individual chromocenter trajectories were tracked for a period of 5 hours and their speed (□m/sec; dashed line) and total path length (□m; continuous line) were quantified. Lines of different color represent different chromocenters.

File name: Supplementary Movie 7
Description: Live-cell-imaging of chromocenter mobility in irradiated SN oocytes. Composite image of confocal microscopy (40X) following microinjection of a fluorescent TALE effector labeling major satellite DNA (green) and recombinant RFP-H2B (red). Individual chromocenter trajectories were tracked for a period of 5 hours and their speed (□m/sec; dashed line) and total path length (μm; continuous line) were quantified. Lines of different color represent different chromocenters.

File name: Supplementary Movie 8
Description: 3D-Rendering of superresolved chromatin structure in control wild-type oocytes. 3D-surface-renderings of individual chromocenters were generated using IMARIS and are shown in green, large-scale chromatin fibers corresponding to euchromatin regions are shown in red.

File name: Supplementary Movie 9
Description: 3D-Rendering of superresolved chromatin structure in irradiated wild-type oocytes. 3D-surface-renderings of individual chromocenters were generated using IMARIS and are shown in green, large-scale chromatin fibers corresponding to euchromatin regions are shown in red. Note the striking chromocenter decondensation at 24 h following irradiation (5 Gy).

File name: Supplementary Movie 10
Description: 3D-Rendering of superresolved chromatin structure in senescent oocytes. Representative example of a senescent 10-month-old oocyte, 3D-surface-renderings of individual chromocenters were generated using IMARIS and are shown in green, large scale chromatin fibers corresponding to euchromatin regions are shown in red. Note the striking chromocenter decondensation at chromocenters.
